# Micro- and Macronutrients in Endometrial Cancer—From Metallomic Analysis to Improvements in Treatment Strategies

**DOI:** 10.3390/ijms25189918

**Published:** 2024-09-14

**Authors:** Gabriela Hunek, Julita Zembala, Jacek Januszewski, Aleksandra Bełżek, Kinga Syty, Zoulikha Jabiry-Zieniewicz, Artur Ludwin, Jolanta Flieger, Jacek Baj

**Affiliations:** 1 Chair and Department of Forensic Medicine, Medical University of Lublin, Jaczewskiego 8b, 20-090 Lublin, Poland; hunekgabriela@gmail.com; 2 First Department of Obstetrics and Gynecology, Medical University of Warsaw, Starynkiewicza 1/3, 02-015 Warsaw, Poland; zembalajulita@gmail.com (J.Z.); zoulikha.jabiry-zienkiewicz@wum.edu.pl (Z.J.-Z.); ludwin@cm-uj.krakow.pl (A.L.); 3 Department of Correct, Clinical and Imaging Anatomy, Chair of Fundamental Sciences, Medical University of Lublin, Jaczewskiego 4, 20-090 Lublin, Poland; jacek.januszewski000@gmail.com (J.J.); o.belzek@gmail.com (A.B.); 4 Institute of Health Sciences, John Paul the II Catholic University of Lublin, Konstantynów 1G, 20-708 Lublin, Poland; kinga.syty@kul.pl; 5 Department of Analytical Chemistry, Medical University of Lublin, Chodźki 4A, 20-093 Lublin, Poland; jolanta.flieger@umlub.pl

**Keywords:** micronutrients, macronutrients, uterine cancer, metals, metallomic analysis, trace elements, metallomics, vitamins

## Abstract

Endometrial cancer is reported to be one of the most prevalent cancers of the female reproductive organs worldwide, with increasing incidence and mortality rates over the past decade. Early diagnosis is critical for effective treatment. Recently, there has been a growing focus on the role of nutrition and micronutrient and macronutrient status in patients with gynecologic cancers, including endometrial cancer. In the following paper, we have conducted an in-depth narrative literature review with the aim of evaluating the results of metallomic studies specifically concerning the micro- and macronutrient status of patients with endometrial cancer. The main objective of the paper was to analyze the results regarding the nutritional status of endometrial cancer patients and describe the role of chosen elements in the onset and progression of endometrial carcinogenesis. Further, we have focused on the evaluation of the usage of the described elements in the potential treatment of the abovementioned cancer, as well as the possible prevention of cancer considering proper supplementation of chosen elements in healthy individuals. Calcium supplementation has been proposed to reduce the risk of endometrial cancer, although some studies offer conflicting evidence. Deficiencies in phosphorus, selenium, and zinc have been inversely associated with endometrial cancer risk, suggesting they may play a protective role, whereas excessive levels of iron, copper, and cadmium have been positively correlated with increased risk. However, the molecular mechanisms by which these elements affect endometrial carcinogenesis are not fully understood, and current findings are often contradictory. Further research is needed to clarify these relationships and to evaluate the potential of nutritional interventions for the prevention and treatment of endometrial cancer.

## 1. Introduction

Corpus uteri cancer, or endometrial cancer, is the cancer of the inner epithelial lining of the uterus with an increasing prevalence in high-income countries, with 417,367 new cases reported worldwide in 2020 [1]. It is also the sixth most commonly diagnosed cancer in women globally and fourth in the United States [1]. Most cases of endometrial cancer occur between the ages of 65 and 75 [2]. Racial disparity, socioeconomic status, and geographical differences significantly influence the incidence and mortality of endometrial cancer. According to GLOBOCAN 2020 data, the highest incidence rates are observed in North America, Europe, Polynesia, Australia, and New Zealand, whereas the lowest rates are found in most African regions and South-Central Asia [1]. In terms of mortality, there were 97,370 deaths in 2020, with North America having the highest number of fatalities. While 67% of patients are diagnosed with early-stage disease, which has an 81% 5-year overall survival (OS) rate, the 5-year OS rates for stage IVA and IVB endometrial cancer drop to 17% and 15%, respectively [3].

The risk of endometrial cancer increases with age, certain ethnicities, higher body mass index (BMI), and prolonged exposure to endogenous or exogenous estrogen [4]. Obesity is a major risk factor for endometrial cancer, especially in high-income countries where both obesity rates and physical inactivity are prevalent [5]. Additional risk factors include the use of tamoxifen in the treatment and prevention of breast cancer, early menarche (before age 12), late menopause (after age 55), lower parity, metabolic syndrome, family history of endometrial cancer, and genetic predisposition, such as Lynch Syndrome [5]. Conversely, a lower risk of endometrial cancer is associated with maintaining a normal BMI, having a higher number of pregnancies, and using oral contraceptives [4,5].

Endometrial cancer is categorized into two principal subgroups: type I and type II [6,7,8]. Type I endometrial cancer, which comprises approximately 80–90% of cases, tends to be less aggressive. Histologically, these tumors are often adenocarcinomas, with or without squamous differentiation, and are typically well-differentiated [9]. Epidemiological studies indicate that the carcinogenesis of type I endometrial tumors follows a multistep process beginning with simple endometrial hyperplasia, advancing to complex atypical hyperplasia, and subsequently developing into endometrial intraepithelial neoplasia (EIN), a precursor lesion [7,8]. Conversely, type II endometrial cancer, accounting for only 10–20% of cases, exhibits markedly higher aggression and is responsible for at least 40% of endometrial cancer-related mortalities [8]. The histological subtypes within type II endometrial cancer include uterine papillary serous carcinoma (UPSC) and clear cell carcinoma, both of which seem to originate from an atrophic endometrium and progress to the precursor lesion known as endometrial glandular dysplasia (EmGD) [8,10].

Among women in the perimenopausal and postmenopausal stages, postmenopausal bleeding (PMB) is a prevalent reason for gynecological consultations, accounting for about two-thirds of visits [11,12]. PMB serves as a significant indicator of potential endometrial cancer cases, with approximately 90% of endometrial cancer patients experiencing this symptom [12]. However, it is noteworthy that PMB leads to an actual endometrial cancer diagnosis in only about 9% of these cases [13]. While abnormal uterine bleeding remains the primary presenting symptom of endometrial cancer, it may be accompanied by other signs such as vaginal discharge and the presence of pyometra, a uterine infection, in certain cases [11].

Moreover, patients with advanced endometrial cancer may manifest symptoms akin to those seen in advanced ovarian cancer [11]. These symptoms include abdominal pain, abdominal distension, and alterations in bowel habits, such as constipation or diarrhea. This overlap in symptoms underscores the importance of comprehensive diagnostic evaluation, especially in cases where endometrial cancer and ovarian cancer share similar clinical presentations.

Trace elements play crucial roles in fundamental metabolic processes, serving as essential components of enzymatic reactions and contributing to the functioning of proteins and transcriptional factors [14]. Essential trace elements in the human body include zinc (Zn), copper (Cu), selenium (Se), chromium (Cr), cobalt (Co), iodine (I), manganese (Mn), and molybdenum (Mo) [14]. Disruptions in the balance of trace minerals can arise from various factors such as hereditary disorders, kidney dialysis, parenteral nutrition, or restrictive dietary patterns [14]. These disturbances may lead to micronutrient deficits or exceeding safe levels of certain elements, resulting in the dysregulation of cellular functions due to the impairment of enzymatic activity, leading to negative health conditions.

The objective of this review is to provide an overview of current understanding regarding the variations in trace element concentrations associated with endometrial cancer. The latest developments in biochemistry have sparked interest among scientists in exploring a new area, particularly focused on influence of trace elements in cancer development and synthesizing metal complexes capable of mimicking the functional features of natural metalloproteins.

## 2. Micronutrients

### 2.1. Calcium

Estrogen is a crucial hormone that quickly triggers the movement of calcium (Ca) and controls the expression of Ca-related proteins in the endometrium [15,16]. Continuous exposure to estrogen without the counteracting effects of progesterone significantly raises the likelihood of developing endometrial cancer. Estrogen-dependent cases account for 75–90% of all endometrial cancers [17]. The presence of estrogen triggers the entry of calcium into cells, and the increased calcium levels rapidly initiate a cascade of responses. Any disruption in this mechanism has the potential to contribute to cancer development and advancement. Frequently, the sudden increase in Ca^2+^ levels triggers the process of photophosphorylation in kinase cascades and controls transcription factor activity, thereby impacting cellular processes and function [18]. Furthermore, excessive accumulation of calcium ions in the mitochondria may result in an increase in mitochondrial reactive oxygen species (ROS), facilitating tumor development, cancer progression, and metastasis [19]. Upon treatment of endometrial cancer cells with estrogen, there was an observed increase in the expression of estrogen-related receptors γ (ERRγ) and S100 calcium binding protein A4 (S100A4), and a positive correlation was found (R = 0.886, *p* = 0.0079) [20]. S100A4 plays an important role in promoting tumor invasion by initiating the epithelial–mesenchymal transition. This process forms a more intrusive cellular phenotype and alters the expression of numerous fundamental molecules, including E-cadherin [21].

When exposed to 17β-estradiol (E2), normal endometrial epithelial cells take in Ca^2+^ from their surroundings [22]. Nevertheless, when RL95-2 cells are placed in a calcium-free environment outside the cell, 17β-estradiol does not induce calcium variations. This suggests that these cells depend on calcium from external sources. However, if the intracellular calcium stores are exhausted, the increase in calcium levels caused by 17β-estradiol is significantly enhanced, while the administration of a protein kinase C (PKC) inhibitor diminishes the response. This indicates that the release of calcium from within the cell through the PKC-sensitive pathway plays a role in the elevation of intracellular calcium caused by 17β-estradiol [15]. Nifedipine, a calcium channel blocker, effectively suppresses the fast calcium influx in endometrial cancer cells caused by estrogen [15].

Cellular studies have discovered that lipids play a direct and indirect role in regulating cytosolic Ca^2+^ and Ca^2+^-mediated signaling in cancer cells. Lipids facilitate the advancement of cancer through Ca^2+^ influx through membrane channels [23]. Research has demonstrated that the utilization of lipid-lowering medications, specifically statins, has a positive impact on endometrial cancer and cytosolic Ca^2+^ levels. Long-term use of statins has been found to decrease the incidence of endometrial cancer and enhance survival rates [24,25].

The disruption of calcium channels plays a crucial role in the complex development of uterine cancer. Endometrial cancer elevates the expression of *CACNA1D*, a type of voltage-gated calcium channel (VGCC). This stimulates cell growth and movement and triggers programmed cell death in Ishikawa cells, as well as the entry of calcium ions caused by estrogen [26]. Mibefradil is a substance that inhibits T-type VGCC and has been found to decrease the survival of Hec-1A cells and promote the activation of proapoptotic proteins [27]. Long-term usage of calcium channel blockers (CCB) may contribute to the development of postoperative lymph cysts. Additionally, CCB usage leads to an extended period of pelvic drainage in patients with cervical cancer and endometrial carcinoma who have undergone pelvic lymphadenectomy [28]. In vitro, the overexpression of *CACNA2D3* significantly suppressed the growth and migration of cells while also enhancing apoptosis and the entry of calcium into the cells. The tumor growth in an in vivo xenograft model was effectively inhibited by inducing its overexpression using lentiviral particle injection [29]. Both in vivo and in vitro observations linked the advancement of cancer to the presence of elevated levels of transient receptor potential vanilloid 4 (TRPV4). TRPV4 activation induces increased calcium influx, subsequently triggering the RhoA/ROCK1 pathway, causing changes in the actin cytoskeleton and facilitating cell migration [30]. Pharmacologically inhibiting TRPV4 with HC067047 or reducing the presence of TRPV4 has been found to effectively prevent metastasis, as demonstrated in glioma cells. This discovery suggests that these methods could be repurposed for the treatment of endometrial cancer [30,31]. The reduction of Transient Receptor Potential Melastatin 4 (TRPM4) stimulates endometrial cancer cell growth and metastasis. The results of the gene set enrichment analysis showed a positive correlation between high *TRPM4* expression and both early and late estrogen responses. Estrogen can initially enhance TRPM4 production; however, chronic exposure to estrogen or its subsequent effects can result in a decrease in *TRPM4* expression or activity. Therefore, the findings finally indicated that the level of TRPM4 content diminished as estrogen levels increased. The estrogen receptor antagonist ICI182780 was found to inhibit these effects, suggesting that membrane estrogen receptors may play a role in these processes [32]. Another possible therapeutic target for endometrial cancer is the transient receptor potential cation channel subfamily V member 2 (TRPV2). Overexpression of *TRPV2* has been found to enhance the cytotoxic effect of cisplatin in Ishikawa cells [33]. Co-administering a TRPV4 activator, such as cannabidiol, with cisplatin can improve the targeted distribution of cytotoxic drugs to the tumor site and effectively inhibit tumor development [34]. The breakdown of the extracellular matrix (ECM) is crucial for the invasion of cancer cells and distant metastasis. Calcium ions entering through TRPV2 channels can increase the expression of certain invasive enzymes, including matrix metalloproteinases and cathepsin B. These enzymes can break down the ECM and create an environment conducive to cancer invasion [35]. 

A 2008 systematic review examined five case-control studies that investigated the correlation between dietary and supplemental calcium. The review found that the results were mainly inconclusive, but there was some evidence suggesting some possible protective effects of calcium supplements against endometrial cancer [36]. Furthermore, a Greek study found a significant inverse correlation between calcium consumption and the risk of developing endometrial cancer [37]. Conversely, in a cross-sectional study involving 12,437 women, it was found that there is a potential positive correlation between calcium dietary intake and the occurrence of cervical cancer (*p* = 0.026) and endometrial cancer (*p* = 0.034) [38]. The relationship between calcium levels in the body and cancer risk is complex and contradictory. One study indicates a significant decrease in the Ca tissue and serum levels in cervical cancer patients compared to healthy individuals. Researchers attributed this to various factors, including calcium absorption and physiological, dietary, and environmental settings [39]. An alternative study, which included 218 women with cervical cancer, 85 women with endometrial cancer, and 259 healthy women, demonstrated an elevated concentration of calcium in the blood serum in the cervical cancer group. This can be attributed to a potential increase in bone resorption in cervical cancer patients, with no such processes occurring in endometrial cancer patients or healthy controls [40].

### 2.2. Phosphorus

The results of the National Health and Nutrition Examination Survey (NHANES), 2007–2016, involving a sample of 12,437 women aged 20 years and older, indicated that phosphorus dietary intake had a protective effect against cervical cancer and endometrial cancer. After adjusting for covariates, there was a significant inverse association between phosphorus intake and both cervical cancer (*p* = 0.002) and endometrial cancer (*p* < 0.001) [38]. Moreover, recent research has shown that dietary phosphorus plays a protective role in cervical intraepithelial neoplasia (CIN) [41]. This would be consistent with the fact that endometrial cancer survivors were found to have decreased phosphorus intake compared to healthy controls [42]. Contradictorily, previous comprehensive studies have demonstrated elevated phosphorus levels in the blood serum of cancer patients [43]. A 2014 retrospective study found a significant increase in phosphorus levels in blood in cancer patients (mean value 7.80 ± 2.24), regardless of the location of the disease. The phosphorus levels in healthy individuals were within the normal range, with a mean value of 3.38 ± 0.58 [44]. Marshak et al. observed the selective uptake of 32P by cancerous cells as early as 1940 [45]. Papaloucas discovered in 1958 that malignant tissue in cervix uteri carcinoma absorbs almost six times more 32P than normal tissue [46]. Patients who did not respond well to therapy showed a significant absorption of 32P [47]. This highlights the contradictory nature of the findings of phosphorus being potentially protective in dietary intake while elevated phosphorus levels in blood correlated with cancer presence.

Historically, radioactive phosphorus-32 (32P) has been used in the adjuvant therapy of ovarian cancer. In ovarian cancer cases, direct intraperitoneal administration of 32P has been shown to be effective in reducing abdominal tumor incidence while minimizing treatment-related damage [48,49]. Patients diagnosed with endometrial cancer and positive pelvic washings have been treated with intraperitoneal 32P, leading to a reduction in the occurrence of the disease returning [50,51]. In a specific trial, intraperitoneal 32P was administered around 4 weeks post-surgery, followed by vaginal brachytherapy. The 3-year survival statistics for all 27 patients were as follows: 84.2% for OS, 90.7% for cause-specific survival, and 74.4% for disease-free survival [52]. The study by Fakiris et al. examined the effectiveness of intraperitoneal 32P and vaginal brachytherapy as therapeutic interventions for uterine papillary serous carcinoma and clear cell carcinoma. The trial only included individuals with negative pelvic and peri-aortic nodes [53]. The study found that the adjuvant therapy was well-tolerated, with minimal low-level toxicity and no moderate-to-severe toxicities (grades 2, 3, or 4). Additionally, the study emphasized the importance of vaginal cuff brachytherapy in reducing the occurrence of vaginal recurrences, as the limited tissue penetration of 8 mm by 32P, a beta emitter, prevents it from delivering an adequate dosage to the vaginal cuff [53,54].

Black phosphorus nanosheets are considered a highly promising nanocarrier for the simultaneous application of chemotherapy and photothermal therapy. A layer of polydopamine was applied to the nanosheets in order to form a novel nanocapsule. The nanocapsule was further altered by including a targeting polymer comprising a mercapto group, polyethylene glycol, and folic acid. The nanocapsule included doxorubicin, a typical medication utilized in the treatment of cervical cancer. The drug delivery system utilizing black phosphorus exhibited enhanced stability, notably high photothermal efficiency, and the capacity to specifically target cancer cells [55].

### 2.3. Sodium

Sodium butyrate (NaBu) exhibits tumor-suppressing properties in both in vitro and in vivo models of endometrial cancer [56]. Researchers demonstrated that NaBu decreased the ability of rat endometrial cells to renew themselves and entirely inhibited the development of colonies on soft agar. This suggests that the treatment caused the production of reactive oxygen species (ROS) within the cells and resulted in DNA damage [57]. Sodium butyrate can increase the production of RBM3 and decrease the production of SLC7A11 in a roundabout way, which starts ferroptosis. This mechanism has the potential to be a cancer treatment approach. The results showed that Ishikawa and HEC-1B cells were less likely to survive when NaBu was applied at a concentration of 5 mM for 24 h. The effect was different depending on how long the cells were exposed to it. NaBu suppressed the colony-forming, proliferative, migratory, and invasive capacities of Ishikawa and HEC-1B cells [56]. In endometrial cancer cells, NaBu activated the G2/M phase arrest. RBM3 is an extensively involved RNA-binding protein that plays a crucial role in several post-transcriptional modification processes, including RNA splicing, RNA transport, RNA sequence editing, RNA intracellular localization, and translation. It accomplishes this by binding to RNA and interacting with it [58,59,60,61,62]. *SLC7A11* is responsible for encoding an essential element of the cystine/glutamate transporter. This transporter facilitates the movement of cystine into cells, which in turn promotes the production of glutathione and improves the effectiveness of the cell’s antioxidant system [63]. The suppression of *SLC7A11* gene expression and disruption of glutathione metabolism through NaBu therapy induce ferroptosis in endometrial cancer cells. This suggests that the signaling pathway involving NaBu, RBM3, and SLC7A11 could be a potential therapeutic target for the detection and treatment of endometrial cancer [56]. The primary choice for treating cervical cancer is widely considered to be cisplatin-based chemotherapy. Nevertheless, certain patients may exhibit a negative prognosis as a result of their resistance to chemotherapy. Therefore, Chu et al. conducted a study on the potential benefits of combining sodium butyrate with chemotherapy [64]. Sodium butyrate, an inhibitor of histone deacetylases (HDACs), has shown promise as a sensitizer of cancer cells to cisplatin [65]. NaBu, in combination with cisplatin, effectively suppressed the growth of cervical cancer cells by inducing apoptosis. In vivo tests demonstrated that pairing NaBu with cisplatin effectively suppressed tumor development and triggered necrosis in cancer cells. A single application of NaBu triggered the Wnt signaling pathway and caused partial epithelial–mesenchymal transition (EMT). The co-administration of cisplatin and NaBu effectively suppressed cellular migration and invasion by disrupting the nuclear translocation of β-catenin, reversing EMT, and decreasing the expression of *MMP2*, *MMP7*, and *MMP9*. Scientists have found that sodium cantharidinate targets *PTPN1* specifically. This stops the PI3K/AKT pathway and makes cervical cancer cells more vulnerable to cisplatin. Protein tyrosine phosphatases (PTPs) remove phosphate groups from tyrosine residues, thereby modulating signal transduction to influence biological processes such as cell proliferation. This investigation focused on targeting PTP nonreceptor type 1 [66]. Cancer cells are widely known for their heightened glucose absorption and aerobic glycolysis [67]. Consequently, selectively blocking the absorption of glucose in tumors while preserving the normal functioning of unaffected organs (such as the heart, muscle, and brain) poses a significant therapeutic obstacle. However, in cancers that absorb glucose through SGLTs, reducing SGLT function may significantly reduce sugar uptake and cell proliferation [68,69]. LEFTY2, a cytokine secreted in the premenstrual period, acts as a suppressor of cell proliferation and tumor development. LEFTY2 reduces the activity of the Na^+^/H^+^ exchanger, leading to a decrease in glycolytic flux and lactate generation in endometrial cancer cells. LEFTY2 facilitates the conversion of stored glucose into glycogen. Normal endometrial cells showed no such effects [70]. The voltage-gated sodium channels are pivotal in facilitating rapid action potentials in nerve and cardiac conduction [71]. Recent discoveries have revealed their significant involvement in the development and progression of cancer [72]. The transcriptional levels of all Na α and β subunits were assessed using real-time PCR in endometrial cancer. The analysis included both carcinoma tissues and surrounding nonneoplastic tissues. Among the NaV subtypes, Nav1.7 exhibited the highest expression in endometrial cancer tissues. The expression of Nav1.7 was strongly correlated with tumor size and local lymph node metastasis, as well as the 5-year and 10-year survival rates. The Nav1.7 blocker PF-05089771 inhibits this channel, leading to cancer cell death and reducing cancer cell invasion. The study investigated the relationship between Nav1.7 tumor expression and the clinical outcome of endometrial cancer patients. It was observed that patients with high-level tumor expression of Nav1.7 had a significantly lower 5-year and 10-year survival rate compared to the Nav1.7-low group (38% vs. 81% and 19% vs. 62%, respectively). The study observed that 75% of endometrial cancer cases showed an increased presence of Nav1.7 in tumor tissue. The levels of the Nav1.7 α subunit in endometrial cancer samples were found to be around 25 times greater than in nonneoplastic tissue biopsies [73]. Quantitative real-time PCR analysis demonstrated that the mRNA levels of the NaV1.6 a-subunit in cervical cancer samples were approximately 40 times greater than in noncancerous cervical biopsies. In cervical cancer, an a-subunit variant of NaV1.7 also showed a significant increase in mRNA levels, almost 20 times higher than normal [74]. Furthermore, the NaV1.6 protein exhibited a clear difference in its distribution within cancer cells compared to non-cancer cells, indicating that these sodium channels are relocated in the plasma membrane in association with cancer [74,75]. In in vitro experiments, the inhibition of voltage-gated sodium channels using tetrodotoxin and Cn2 did not have an impact on the proliferation or migration of cervical cancer primary culture cells. However, it did result in a reduction of around 20% in the invasiveness of these cells. The findings suggest an increase in NaV1.6 in cervical cancer, which could potentially serve as a new molecular marker to track the metastatic behavior of this type of carcinoma. Furthermore, the development of new therapeutic approaches could potentially target it [74].

The voltage-gated sodium channel is a significant factor in the pathogenesis of cervical cancer. However, the clinical and pathological implications of the epithelial sodium channel have not been investigated. Song et al. conducted a study with the aim of finding the dysregulation of genes that encode ENaC; specifically, *SCNN1A*, *SCNN1B*, and *SCNN1G. SCNN1A*, *SCNN1B*, and *SCNN1G* have higher expression levels in normal tissues compared to tumor tissues. These genes are functionally involved in regulating salt and water balance. The survival study demonstrated that the concurrent overexpression of all three genes was linked to improved OS and a lower histologic grade of the tumor [76]. Tang et al. used radioiodine therapy for cervical cancer by utilizing the *early growth response* (*Egr-1*) promoter to regulate the expression of the *sodium-iodine symporter* (*hNIS*) gene [77]. The findings indicate that using the *Egr-1* promoter to control *hNIS* can greatly increase iodine absorption and retention in cancer cells. Radiation further strengthens this effect, creating a positive feedback loop that could enhance radionuclide therapy in vivo.

### 2.4. Potassium

Potential negative correlations between potassium intake and endometrial cancer have been identified in studies (*p* = 0.032) and (*p* = 0.007) [38,78]. Additionally, it has been discovered that potassium can have a protective effect against CIN^2+^ at specific dosages below the average total consumption. However, the exact mechanism behind the association between potassium and CIN necessitates further examination [41,79,80]. 

The precise method by which voltage-gated potassium (Kv) channels contribute to cancer cell migration remains unclear [81]. Eag1 (ether a′-go-go-1, KCNH1, Kv10.1) is a specific form of voltage-gated potassium channel that shows restricted expression in healthy tissues, but its presence has been detected in several types of tumors, including cervical cancers [82]. The work conducted by Sim et al. provided evidence that the Kv3 blocker BDS-II effectively suppressed the migration of cancer cells [83]. The Kv3 inhibitor also deactivated the AKT pathway in HeLa cells and affected the protein vimentin, which plays a role in promoting and regulating cell migration. Cancer cell migration is regulated by vimentin through the involvement of many signaling pathways, such as the AKT pathway. Furthermore, the abundance of vimentin in cancer is associated with malignancy [84,85,86,87,88]. Vimentin is a downstream target of AKT; AKT induces vimentin phosphorylation, and AKT inhibition evokes vimentin proteolysis by caspase-3 [87,89,90]. The inhibition of AKT leads to a decrease in vimentin and Kv3.4 levels, resulting in the suppression of cell migration in HeLa cells. 

Guadalupe Chávez-López and colleagues conducted a study to examine the impact of astemizole on cervical cancer cells [82]. The results showed a significant reduction in cell growth by 40% and a substantial rise in apoptosis in all the cell lines investigated. Cervical cancer is a complex disease that is influenced by multiple factors, with the main element contributing to its development being infection with the human papillomavirus (HPV). HPV oncogenes and p53 have a regulatory effect on *Eag1* expression [91,92]. Astemizole reduces the metabolic activity of normal keratinocytes that have been permanently modified to contain the *E6/E7* HPV oncogenes [91]. The aforementioned study observed the antiproliferative and proapoptotic effects of astemizole in cervical cancer cell lines with different HPV statuses [82]. 

Cervical cancer is a very suitable malignancy to be targeted by calcitriol. Compared to healthy cervical tissue, cervical carcinomas have a greater fraction of the vitamin D receptor (VDR) and the anabolic and catabolic vitamin D hydroxylases [93]. In addition, cervical cancer has high levels of *Eag1* expression, which is further increased by cervical cancer risk factors such as estrogens and HPV oncogenes [91,94]. The ongoing trials, in which the calcitriol synthesis enzyme CYP27B1 is overexpressed, provide compelling evidence that increased calcitriol synthesis from its precursor 25OHD3 suppresses the expression of the *Eag1* gene. Therefore, modifying one’s diet or increasing sun exposure, which leads to the production of more calcitriol precursors, may assist in decreasing *Eag1* expression [95]. 

Research has demonstrated that potassium channels regulate the course of the cell cycle, leading to an increase in cell proliferation. Additionally, they stimulate changes in the cytoskeleton to boost the ability of cells to invade and migrate. Tetraethylammonium (TEA), a broad-spectrum antagonist of various types of potassium channels, inhibits the formation of colonies in endometrial cancer cells by suppressing putative tumor-initiating cells (TIC). The findings collected by Schickling et al. also demonstrated that discontinuation of TEA leads to a substantial increase in carcinogenesis [96]. TEA modifies the ability of tumors to form by suppressing the formation of subgroups of cells that have a high level of ALDH1 activity and a low basal proliferation rate. Cancer patients in remission, in particular those who have a high risk of recurrence, may benefit from such therapy to prolong disease-free survival. 

According to Huang et al., voltage-gated potassium channel subfamily A member 1 (KCNA1/Kv1.1) was significantly higher in cervical cancer tumor tissues compared to surrounding non-tumor tissues; KCNA1 functions as a potassium-selective channel that allows the passage of potassium ions across the electrochemical gradient [97]. This process is crucial for the repolarization of the membrane [98]. *KCNA1* modulates the mitochondrial capacity of HeLa cells. Research has demonstrated that alterations in mitochondria during apoptosis are associated with the concentration of potassium ions within cells, which controls the size of mitochondria and the production of reactive oxygen species [99]. Deviation from normal intracellular potassium ion concentration can disrupt the transmembrane potential of the mitochondrial inner membrane and trigger the release of Cytochrome C [100]. In addition, *KCNA1* may possibly contribute to the process of programmed cell death in cervical cancer cells. The researchers found a significant correlation between high levels of *KCNA1* expression and shorter survival durations, indicating a negative prognosis. Hence, the assessment of *KCNA1* expression levels in cervical cancer can serve as a prognostic indicator for tumor stages and patient survival durations [97]. 

K^+^-Cl^−^ cotransporters (KCCs) have a crucial function in the operation of epithelial cells, contributing to the maintenance of ionic balance and influencing cell shape, cell division, and movement. The isoform *KCC2* exhibits broad expression in various human cancer cell lines, such as the SiHa cervical cancer cell line, and is not limited solely to neurons. KCC2 promotes the advancement of cervical cancer through a mechanism that is not dependent on ion transport. It actively contributes to the malignant characteristics of cervical cancer cells by enhancing IGF-1-induced cell migration and invasion. Overexpression of *KCC2* did not have a significant impact on cell proliferation. However, it greatly inhibited cell spreading and the organization of stress fibers. Conversely, the knockdown of *KCC2* had the opposite effects [101]. 

Scarth et al. establish that pharmacological agents that activate or inhibit ATP-sensitive potassium ion (KATP) channels are active in HPV-positive cells and that this activity is essential for the production of HPV oncoproteins. Suppressing KATP channels, either by reducing the expression of specific subunits using siRNA or by utilizing approved inhibitors to prevent their activity, greatly hinders cell growth and advancement through the cell cycle. HPV can enhance the activity of KATP channels by increasing the expression of the SUR1 subunit through the action of the E7 protein. This effect is shown in both cervical disease and cervical cancer tissue. KATP channels control the G1/S phase transition, which is responsible for promoting cell growth [102]. Consequently, the suppression of KATP led to a rise in the percentage of cells in the G1 phase and, correspondingly, a decline in the production of cyclin D1 and E1. This aligns with previous findings for other types of cells [103,104,105]. During the G1-S phase checkpoint, cells experience rapid hyperpolarization, which is primarily facilitated by K^+^ efflux channels [106,107].

### 2.5. Magnesium

Animal development and the process of cell differentiation recognize *magnesium transporter subtype 1* (*MAGT1*). The study showed that S-phase arrest and apoptosis significantly hindered cell proliferation in the *MAGT1*-knocked-down cells (HeLa and SiHa cells treated with different siRNAs). The elevated levels of p21, cyclin-A1, and cyclin-B1, along with the reduced levels of MYC, cyclin-D1, cyclin-E1, and CDK2, further supported this. The bioinformatics research revealed a significant association between *MAGT1* and the MAPK signaling pathway. Western blot examination verified that the phosphorylation of extracellular signal-related protein kinase 1/2 (ERK1/2) and p38 was significantly decreased in the groups where *MAGT1* was down-regulated. Viral proteins E6 and E7 require *MAGT1* to function, facilitating cell division and transitioning from the G1 to the S phase of the cell cycle. 

Thus, *MAGT1* is essential for the growth and multiplication of cervical cancer cells that are positive for the human papillomavirus (HPV). It also plays a key role in facilitating the progression of the cell cycle during the S-phase and regulating the ERK/p38 MAPK signaling pathway. The data suggest that *MAGT1* has the potential to be a new target for anticancer research [108]. For seven days, administering hydralazine and magnesium valproate (HV) to individuals with cervical cancer resulted in the up-regulation of 964 genes. The ribosome protein and oxidative phosphorylation pathways had the highest number of up-regulated genes. The following were MAPK signaling, tight junction, adherens junction, actin cytoskeleton, cell cycle, focal adhesion, apoptosis, proteasome, Wnt signaling, and antigen processing and presentation pathways. Furthermore, there was a reported elevation in acetylated p53 levels. Using HV in epigenetic therapy activates genes and acetylates proteins in the primary tumors of cervical cancer patients. Several of these reactivated genes have a clear function as tumor suppressors. The overall gene expression pattern elicited by HV indicates that this therapy affects pathways associated with energy generation, perhaps facilitating apoptosis. In cancer therapy, the justification for employing epigenetic medicines, including DNA methylation and HDAC inhibitors, has been based on the notion that rectifying epigenetic abnormalities will activate tumor suppressor genes and therefore have anti-tumor effects [109]. 

In a separate trial, twelve recently diagnosed patients with cervical cancer received treatment with magnesium valproate [110]. The patients were divided into four groups, with four patients in each group, and each group received a different dose level of the medication. The recommended dosage is 20 mg/kg, 30 mg/kg, or 40 mg/kg for a duration of 5 days administered orally. Following treatment, an increase in H3 and H4 acetylation levels were observed in the tumors of nine and seven patients, respectively. Additionally, six patients exhibited increased acetylation levels in both histones. The serum concentrations of valproic acid varied between 73.6 and 170.49 µg/mL. In eight individuals, the tumor’s deacetylase activity decreased, accounting for 80% of the cases, whereas two patients had either no change or a slight increase. There was a significant change in HDAC activity between the data before and after therapy (mean, 0.36 vs. 0.21, *p* < 0.0264, two-tailed *t*-test). There was no association between the hyperacetylation of H3 and H4 tumors and the amounts of valproic acid in the serum. Magnesium valproate, administered at a dosage ranging from 20 to 40 mg/kg, effectively suppresses deacetylase activity and causes excessive acetylation of histones in tumor tissues. 

Magnesium is essential for people undergoing chemotherapy. The study examined the nephroprotective effects of administering magnesium for hydration in patients with cervical cancer who were undergoing treatment with cisplatin. In the non-Mg-hydration group, the blood creatinine level increased significantly from 0.58 to 0.75 mg/dL, while the estimated glomerular filtration rate decreased significantly from 85.1 to 66.5 mL/min due to chemotherapy. However, there were no significant changes observed in the Mg-hydration group. Among patients with cervical cancer undergoing chemotherapy with cisplatin alone, a magnesium dose of 15 mEq was determined to have nephroprotective benefits [111].

### 2.6. Zinc

Researchers have linked the development of cancer to zinc, a crucial trace element that plays a key role in maintaining balance within the body [112]. Zinc (Zn) plays a significant role in maintaining the stability of DNA and RNA within cells. Zinc supplementation improves DNA synthesis, whereas zinc depletion inhibits it. Copper and zinc serve as co-factors in the metabolic reactions facilitated by superoxide dismutase (SOD) [113,114]. SOD is recognized for its function in safeguarding tissues from damage caused by free radicals and in preventing the onset of cancer [115]. 

Zn deficiency may result in a decrease in the function of Zn-dependent proteins that maintain DNA integrity. This lack of Zn could also increase the production of the tumor suppressor protein p53 but hinder its ability to bind to DNA, as well as the ability of nuclear factor kB (NFkB) and AP-1 transcription factors to bind to DNA [116]. These findings indicate that a reduction in cellular zinc levels leads to DNA damage and hinders the systems that respond to DNA damage. This compromises DNA integrity, potentially increasing the risk of developing cancer [117]. 

Zinc plays an important role in promoting cell growth and preserving cell membrane structure [118]. Consequently, cancer cells may uptake zinc from the bloodstream to sustain their growth and maintain the integrity of their membranes. The malignant cell has the ability to use the circulating Zn to promote tumor growth and preserve membrane integrity [119]. This could be the potential cause of the reduction in zinc levels in cancer patients [120]. Zn has an anticarcinogenic effect on DNA synthesis, RNA transcription, immune system functions, cell division, and growth processes [121,122]. 

Patients diagnosed with endometrial cancer had significantly lower zinc levels (mean 1.83 ± 0.71 mg/L) compared to the control group (mean 2.48 ± 0.89 mg/L) (*p* < 0.001) [123]. The findings of a meta-analysis of twelve publications examining serum zinc levels and cervical cancer indicate that individuals with cervical cancer had considerably lower levels of serum zinc compared to individuals without cervical cancer. Geographic stratification of the data revealed the possible association between elevated serum zinc levels and protective factor against cervical cancer in Asian women [124]. 

Patients with gynecologic malignancies who underwent chemotherapy experienced a reduction in their serum zinc levels and had a tendency to develop changes in their sense of taste. However, the use of zinc supplements increased the serum levels without any significant problems and may have averted any changes in taste perception [125]. Concerning the consumption of zinc in one’s diet, the results are inconclusive, as one study did not find a correlation between dietary zinc and the risk of cervical disease. However, a second investigation discovered that dietary zinc decreased the incidence of CIN, whereas insufficient zinc consumption was identified as a risk factor for cervical cancer [126,127]. 

There was a negative and independent association between consuming large amounts of zinc in the diet and acquisition of high-risk HPV infection [128]. Barchitta et al. conducted a cross-sectional study, examining 251 Italian women with normal cervical cytology [128]. The study revealed the association between a higher zinc and dietary antioxidant intake and reduced likelihood of contracting high-risk human HPV (OR = 0.46; 95% CI = 0.27–0.80; *p*-value = 0.006). An OR of 0.46 indicates that women with higher zinc intake were 54% less likely to contract high-risk HPV compared to those with lower intake. 

Prior research has demonstrated that zinc exhibits antiviral activity against a range of viruses, including HIV, HCV, and HPV [122]. Jin et al. provided evidence that zinc citrate significantly enhances the rate of elimination of high-risk HPV [129]. This evidence suggests that Zn may play a significant role in preventing HPV infection. Ayatollahi et al. (2022) randomly assigned 80 women, aged between 21 and 55 years, who were zinc-sufficient and tested positive for HPV with abnormal cervical cytology, into two groups. The first group took oral zinc sulfate tablets (220 mg) every 12 h for a duration of three months (N = 40), while the second group, serving as the control group, did not receive any therapy. 

After a three-month period of monitoring, the likelihood of HPV infection persisting (CI 95% 0.04–0.381; *p* < 0.001) and progressing from the initial cytology results (95% CI 0.777–0.116; *p* = 0.012) was significantly lower. Additionally, this interventional study highlights the significant reduction in the persistence and progression of HPV infection in the zinc-treated group [130]. 

Yang et al.’s study provides evidence that the overexpression of *Retinoblastoma protein-interacting zinc finger gene 1* (*RIZ1*), in conjunction with radiotherapy, enhances apoptosis and induces DNA damage in cervical cancer cells [131]. This suggests that *RIZ1* may function as a tumor suppressor gene. The expression of *RIZ1* typically decreases in cervical cancer tissues. There was a significant correlation between decreased *RIZ1* expression and advanced FIGO stage (*p* = 0.005), deep stromal invasion (*p* = 0.001), lymphovascular space involvement (*p* = 0.041), pelvic lymph node metastases (*p* = 0.005), and postoperative recurrence (*p* = 0.002). The decreased expression of *RIZ1* may play a role in the movement of tumors, their ability to invade surrounding tissues, and the unfavorable prognosis for individuals with cervical cancer. *RIZ1* could serve as a predictive indicator for the prognosis of patients with cervical cancer [132]. 

Mounting data demonstrate that abnormal activation of the epithelial–mesenchymal transition (EMT) is critical in facilitating tumor cell invasion and spread. The EMT facilitates cell detachment and promotes cell mobility, both of which are crucial for the spread of tumor cells. An essential characteristic of EMT is the absence of E-cadherin, which is a protein responsible for cell adhesion. EMT is induced by several transcription factors, with two notable examples being ZEB1 and ZEB2, which are zinc-finger E-box-binding homeobox factors [133,134,135]. Research has linked ectopic overexpression of ZEB1 in breast cancer, ovarian cancer, endometrial cancer, and prostate cancer to inadequate cellular specialization, aggressive disease progression, the formation of secondary tumors, and an unfavorable clinical outlook [136,137,138,139].

### 2.7. Iron

Iron is an essential trace element that plays a crucial role in many biological processes, such as the action of enzymes like catalase, peroxidases, and cytochromes, as well as iron-containing proteins like hemoglobin and myoglobin. However, iron can also act as a potent oxidant, leading to the formation of harmful free radicals, which impact oxidative processes in the body. Therefore, the uptake and transport of iron within the cells of living organisms are tightly controlled to prevent its excessive accumulation and minimize damage caused by oxidative stress. Since there are suspicions that iron may influence the development of estrogen-related cancers, its role in the pathogenesis of these tumors is currently under investigation [140,141].

#### 2.7.1. Diet

The majority of iron in our daily diets comes from non-heme plant-based sources, comprising 80 to 95 percent of intake in typical Western diets. Non-heme iron, present in vegetables and certain animal products, is largely unabsorbed by the body, unlike heme iron, which is readily absorbed and constitutes about 40 percent of the iron found in red meat or its processed forms. While cutting out meat does not significantly affect the total amount of iron consumed, it significantly reduces its absorption. Studies suggest that consuming red meat may increase the risk of endometrial cancer. Although additional iron intake may have little impact on iron stores due to its low absorption, simultaneous intake of heme iron and animal protein significantly increases the absorption of non-heme iron, thereby increasing the potential for oxidative stress-induced damage and DNA damage. The influence of estrogens on the risk of endometrial cancer may outweigh the effects of iron in Western societies, making it difficult to detect any potential effects of iron in the diet, especially heme iron. The positive association between the consumption of iron from animal products and the risk of endometrial cancer may stem from its pro-oxidative properties or modification of estrogenic pro-inflammatory effects in the endometrium. There is evidence suggesting that saturated and monounsaturated fatty acids from animal products may facilitate the transport of iron in its reactive ferrous form (+2) across cell membranes, thereby increasing the oxidative effects of iron in the body. Heme iron, present in red meat, may also activate specific inflammatory pathways, such as the release of tumor necrosis factor (TNF)-α through toll-like receptor 4 (TLR4) activation [141].

A diet rich in total iron and heme iron may lead to increased pro-oxidative burden, which ultimately may contribute to greater oxidative stress and DNA damage. Furthermore, there is evidence that heme iron is positively correlated with the risk of diabetes, obesity, and markers associated with both obesity and diabetes, all of which are suspected or already established risk factors for endometrial cancer. One reason why a statistically significant association is observed between endometrial cancer and the consumption of heme iron and total iron, but not with all individual major sources of iron, such as red meat and processed meat products (e.g., sausages), may be the lower contrast and precision in measuring the etiological factor when assessing only iron intake in the diet and heme iron compared to using overall meat consumption or a specific type of meat as a substitute for iron intake in the diet and heme iron [142].

#### 2.7.2. Iron and Cobalt in Cancer Development

Iron and cobalt are two elements that compete with each other because they often occur in the same oxidation states (2+/3+) and possess similar characteristics. Iron is commonly recognized as a carcinogen in both animals and humans. There is no clear scientific evidence of the potential carcinogenic effects of cobalt, and the existing evidence is mainly based on cell studies conducted on animals. However, both metallic cobalt and soluble cobalt(II) salts belong to the group of carcinogens for which there is evidence from studies on human cells, suggesting their potentially harmful effects [140].

#### 2.7.3. Cervical Cancer

Under the influence of hypoxia induced by CoCl_2_, there is a gradual increase in the expression of glucose transporter 1 (GLUT1) protein depending on the clinical stage of cervical cancer. It has been observed that higher levels of GLUT1 expression correlate with a greater risk of lymph node metastasis. Normal tissue exhibits the lowest expression of this protein, while cervical intraepithelial neoplasia shows higher levels and cervical cancer shows the highest levels. The iron level in cervical cancer tissue is higher than in unchanged tissue, while iron levels in the blood are lower in patients with cervical cancer compared to healthy patients. Additionally, iron levels are higher in cervical cancer tissue than in the case of fibroids [140].

#### 2.7.4. Fibroids

Measurements of iron concentrations in uterine muscle tissue reflect lower levels of iron in the serum of patients with fibroids. Compared to normal and cancerous uterine tissues, iron concentrations in fibroids are significantly lower. Furthermore, compared to other trace elements, differences in iron concentrations show greater specificity for fibroid tissue. The study also revealed higher cobalt levels in the urine of patients with uterine fibroids. There are two main hypotheses explaining these phenomena. Firstly, higher exposure to cobalt may contribute to the growth of smooth muscle cell fibroids. Secondly, uterine fibroids may serve as a site of cobalt accumulation, leading to increased levels of this element in women with this benign tumor compared to those without it [140].

#### 2.7.5. Anemia

It is suggested that oxygen deficiency associated with anemia may disrupt tumor sensitivity to radiation, as cells under hypoxic conditions exhibit two to three times greater resistance to radiation than those in normal oxygen levels. Recent reports suggest that intravenous iron administration may more rapidly improve anemia than oral intake in patients undergoing chemoradiotherapy and may be beneficial for those who have absorption issues or cannot tolerate oral iron intake. There are three main types of iron used for intravenous administration, including iron dextran, iron gluconate, and iron sucrose, with iron sucrose seeming to be the least likely to cause hypersensitivity reactions. In patients undergoing chemoradiotherapy, the mechanism leading to anemia appears to be associated with functional iron deficiency. Often, after prolonged use of erythropoietin, anemia develops, so iron delivery is necessary to maintain adequate red blood cell production. Intravenous iron administration has been shown to more rapidly correct anemia than oral iron tablets, which is beneficial for patients with iron absorption issues or those who do not tolerate oral iron intake. Administration of iron in the form of iron sucrose intravenously has reduced the number of transfusions in patients with anemia in each cycle of chemoradiotherapy for cervical cancer. Therefore, intravenous administration of iron sucrose may be an effective method of preventing anemia in patients with cervical cancer undergoing concurrent chemoradiotherapy [143].

#### 2.7.6. Iron in Uterine Cancer Diagnosis

Sentinel lymph node (SLN) mapping using dyes or radioisotopes has been performed in patients with uterine cancer. Superparamagnetic iron oxide (SPIO) has proven to be safe and effectively taken up by lymph nodes (LNs); however, its accuracy in detecting SLNs in uterine cancer is still unknown. Lymph nodes positive for radioisotopes were also positive for SPIO/MRI in 92% of cases after iron staining and pathological examination. The use of SPIO/MRI and iron staining may be helpful in identifying SLNs and diagnosing lymph node metastases in patients with uterine cancer. Lymph node metastases may interfere with SPIO uptake by disrupting lymph flow and damaging lymph node structure. SPIO can act as a retention-type tracer for SLN mapping as it remains in the lymph nodes. Compared to radioisotopes, SPIO is easier to use and has fewer side effects. Sentinel lymph node diagnosis through iron staining after SPIO administration represents a milestone in pathological diagnosis. Often, it is difficult to precisely locate these nodes during surgery and rapidly conduct pathological examinations. Therefore, evaluating these nodes in postoperative specimens can significantly contribute to accurate diagnosis. Even several days after local SPIO injections, lymph nodes can still be detected through iron staining. It is known that lymph node metastases may disrupt the natural flow of lymph, sometimes hindering the detection of sentinel nodes. In summary, both SPIO and radioisotopes are equally effective in uptake by sentinel nodes in patients with uterine cancer. The use of SPIO/MRI and iron staining may be helpful in identifying sentinel nodes and diagnosing lymph node metastases in these patients [144].

#### 2.7.7. Iron in Endometrial Cancer Development

Endometrial cancer often presents in early stages more frequently than ovarian cancer. It appears that the level of hypoxia-inducible factor 1-alpha (HIF-1α), which increases in the presence of cobalt chloride (II), may be associated with the aggressiveness of endometrial cancer. There is speculation that activation of the classical nuclear factor kappa B (NF-kB) pathway occurs in endometrial cancer cells through HIF-1α. Some studies suggest that the use of iron supplements may be an indicator of endometrial cancer rather than its cause, as higher levels of iron are observed in endometrial tumor tissues compared to healthy tissues (Table 1) [140].

## 3. Trace Elements

### 3.1. Copper

Copper is an essential element that plays a vital role in various biochemical reactions, acting as a cofactor for SOD. This enzyme is crucial for protecting the body against harmful free radicals. The carcinogenic properties of copper are believed to arise from the production of reactive oxygen species, which can damage DNA and initiate the process of angiogenesis in tumors [145]. Additionally, it induces cell apoptosis [146].

#### 3.1.1. Cu as a Serum Marker

Studies indicate a relationship between trace elements and the occurrence of cervical cancer. Levels of six trace elements (Cu, Zn, Fe, Mn, Ca, and Se) were measured in tissues and serum, and the Cu/Zn ratio was determined using atomic absorption spectrophotometry, while selenium content was assessed using atomic fluorescence spectrometry. The results showed that zinc, selenium, and calcium levels in tissues were significantly lower, while copper and iron concentrations, as well as the Cu/Zn ratio, were significantly higher in cervical cancer tissues compared to paired unaffected tissues. In the serum of patients with cervical cancer, zinc, selenium, calcium, and iron levels were lower, while copper and manganese levels, as well as the Cu/Zn ratio, were higher compared to healthy individuals. Copper levels in serum and the Cu/Zn ratio were also significantly higher in patients with cervical cancer compared to the uterine fibroid group. Therefore, elevated copper levels in serum and tissues, along with zinc and selenium deficiencies, may be associated with the development of cervical cancer [39].

Concentrations of Cu and Zn, as well as their ratio (Cu/Zn), in tissues and blood serum may serve as markers in cancer diagnostics. No differences in Cu and Zn levels were observed in uterine fibroid tissues; however, a decrease in Zn levels and an increase in Cu levels and the Cu/Zn ratio were observed in the blood serum of women with uterine fibroids compared to control tissues. This may suggest that copper plays a less significant role in uterine cancer compared to other types of cancer [147]. Despite the lack of a significant correlation between copper levels and age, BMI, multiple pregnancies, and number of births, a positive relationship was observed between zinc levels and the number of births. However, when analyzing patients with endometrial cancer separately, both copper and zinc levels showed a positive correlation with age. The studies suggest that women with endometrial cancer may have altered levels of copper and zinc in their blood serum [123]. It is assumed that low copper levels may increase the risk of cancer by inhibiting the antioxidant action of the enzyme superoxide dismutase, while high copper levels may increase the risk of cancer by catalyzing the formation of free radicals. The generation of free radicals, especially in areas of copper accumulation, is the most likely hypothesis regarding the role of copper in carcinogenesis, as Cu ions bind to cellular proteins. The gradual increase in total Cu concentration in the cell may lead to the formation of nonspecific copper complexes, which are responsible for biological cell damage. Additionally, copper participates in the process of tumor angiogenesis. Studies have shown that copper not only activates angiogenic factors but also has the ability to bind to proteins (such as ceruloplasmin, heparin) that exhibit angiogenic activity only after binding copper ions. Hypotheses have been formulated for both zinc and copper regarding their roles as carcinogenic and anticarcinogenic factors. Elevated copper levels in serum and tissue may be a risk factor, while decreased zinc and selenium levels may contribute to the development of cervical cancer [147].

In contrast to healthy cells, cancer cells have higher levels of copper, which plays a crucial role in the formation of blood vessels. Therefore, targeting copper with special chelating substances in cancer cells can be an effective anti-cancer strategy. The properties of a copper chelator, which is a derivative of tetrazole with a pregnenolone acetyl anion core (ligand-L), were investigated using elemental analysis, ESI-MS, 1H NMR, and 13C NMR. The toxicity of ligand-L on C33A cervical cancer cells was also examined in vitro. It was found that the toxicity of ligand-L arises from the redox reaction of copper, which leads to the generation of reactive oxygen species (ROS), resulting in DNA damage and apoptosis. According to the conclusions of the report, a derivative of tetrazole with a pregnenolone acetyl anion core was developed targeting C33A cells. This substance is designed to act on copper in cancer cells, leading to the induction of cell death through a pro-oxidative process. The outcome of this action is the generation of reactive oxygen species (ROS), which in turn causes DNA damage and apoptosis in cancer cells [148].

#### 3.1.2. Cu with Female Hormones

Research confirms that the development of uterine cancer and fibroids is strongly linked to the action of female hormones such as estrogens and progesterone. There is compelling biochemical evidence for the significant role of estrogens in stimulating the growth of fibroids. Treatment with long-acting gonadotropin-releasing hormone agonists (GnRHs) leads to a reduction in estrogen levels and a decrease in the volume of fibroids. However, analysis of the expression of estrogen receptors (ERs) and progesterone receptors (PgRs) in uterine fibroids provides mixed data. It has been discovered that zinc (Zn) plays a significant role in the “zinc finger” of the alpha estrogen receptor (ER-α), affecting its ability to bind to DNA in response to estrogen. Substituting zinc with copper (Cu) inhibits this binding process, suggesting the significant impact of this interaction on estrogen response regulation [147].

#### 3.1.3. Cu in Intrauterine Devices

Research on the protective effect of intrauterine devices (IUDs) in preventing cancer primarily focuses on devices containing levonorgestrel, especially in the context of endometrial cancer. However, there is also evidence suggesting that both copper intrauterine devices and those containing levonorgestrel may reduce the risk of endometrial cancer. Furthermore, the use of copper intrauterine devices is associated with a decreased risk of cervical intraepithelial neoplasia. Overall, available data suggest that both levonorgestrel-releasing and copper IUDs may decrease the risk of gynecological cancers [149,150]. The use of non-hormonal intrauterine devices, including plastic and copper ones, has been correlated with a reduced risk of endometrial cancer [151].

#### 3.1.4. Copper in Treatment

Metal oxide-based nanoparticles serve as carrier platforms for biologically significant molecules. Copper/TiO_2_ nanoparticles have been thoroughly characterized using various techniques such as UV-Vis spectroscopy, Fourier-transform infrared spectroscopy, differential scanning calorimetry, thermogravimetric analysis, nitrogen physisorption analysis, and scanning electron microscopy. Their biological activity was assessed through DNA degradation analysis and their cytotoxic effect on HeLa cells, which are characterized by cervical cancer. The results suggest that these nanoparticles may potentially be utilized in the therapy of this type of cancer [152].

Cisplatin (DDP) is commonly used as a first-line treatment for solid organ tumors and demonstrates efficacy in the majority of patients with germ cell tumors. Copper transporter 1 (CTR1, also known as hCTR1 encoded by the *SLC31A1* gene) plays a crucial role in copper transport into the cell, which is necessary for transporting cisplatin, likely in the form of reduced Cu(I), into the cell. It is noted that CTR1 significantly increases the internalization fraction of cisplatin in cancer cells. Resistance to cisplatin in tumors is often associated with modifications in the expression level, intracellular localization, or function of CTR1. As the primary copper transporter, CTR1 controls the accumulation of cisplatin in cancer cells. The correlation between higher levels of CTR1 and greater uptake of platinum drugs in cancer cells has been confirmed. Increased expression of CTR1 may sensitize cancer cells to platinum drugs, while decreased expression of CTR1 may lead to resistance. Studies suggest that CTR1, as a membrane protein involved in regulating copper homeostasis, controls the flow of cisplatin and its analogs into cells. It has been shown that in various cervical cancer cell lines, including cisplatin-resistant HeLa cells, CTR1 expression is reduced. Research shows that the C-terminal end of the CTR1 protein is crucial for cisplatin uptake in HeLa cells; HeLa cells overexpressing CTR1 showed significantly greater accumulation of cisplatin. In the case of cisplatin-resistant HeLa/DDP and CaSki/DDP cells, overexpression of CTR1 reduced resistance to cisplatin, while lack of CTR1 expression increased resistance [153].

### 3.2. Chromium

#### 3.2.1. Chromium Assay in Endometrial Cancer Therapy

The antibody-drug conjugate (ADC) sacituzumab govitecan (SG) is designed to target the trophoblast cell-surface antigen 2 (Trop-2), a cell-surface glycoprotein highly expressed in many epithelial cancers. SG delivers the active irinotecan metabolite SN-38 to Trop-2-positive cancer cells. The impact of SG on the viability of primary tumor cell lines was assessed after exposure to SG or control antibodies. Antibody-dependent cell-mediated cytotoxicity (ADCC) against EC cell lines, either positive or negative for Trop-2 expression, was evaluated through 4 h chromium release assays. In a xenograft model, intravenous administration of SG twice weekly for three weeks was well tolerated and showed significant tumor growth inhibition in EC xenografts characterized by poor differentiation and chemotherapy resistance. In summary, SG demonstrated promising preclinical activity against poorly differentiated EC cell lines overexpressing Trop-2 [154].

In the four-hour chromium release assay, cytotoxicity can also be measured against cervical dysplasia cells as well as invasive cervical cancer cells [155].

#### 3.2.2. Uterine Serous Carcinoma (USC)

Uterine serous carcinoma (USC) is an aggressive subtype of endometrial cancer typically associated with poor prognosis. Sacituzumab govitecan (SG) is a novel antibody-drug conjugate (ADC) designed to target the trophoblast cell-surface antigen 2 (Trop-2), a transmembrane calcium signaling protein. SG delivers SN-38, the active metabolite of irinotecan, directly to cancer cells. ADCC against USC cell lines positive or negative for Trop-2 expression was assessed in vitro using 4 h chromium release assays [156].

Expression levels of membrane complement regulatory proteins (mCRPs) CD46, CD55, and CD59 were examined in primary uterine serous carcinoma (USC), and the ability of small interfering RNA (siRNA) targeting these mCRPs to enhance USC sensitivity to complement-dependent cytotoxicity (CDC) and antibody-dependent cell-mediated cytotoxicity (ADCC) induced by trastuzumab was evaluated in vitro. The biological effects of mCRP knockdown by siRNA on USC cell lines overexpressing HER2/neu were assessed through 4 h CDC and ADCC chromium release assays. The study found that uterine serous carcinoma is characterized by high levels of mCRPs CD46, CD55, and CD59. Inhibition using CD55 and CD59 siRNA, but not CD46, increased USC sensitivity to CDC and ADCC in vitro. These results suggest that siRNA targeted to cancer cells could significantly enhance therapeutic efficacy [157].

#### 3.2.3. Uterine Serous Papillary Carcinoma (USPC)

Targeting tissue factor (TF), uterine serous papillary carcinoma (USPC) represents a particularly aggressive type of endometrial cancer. The immuno-conjugate molecule hI-con1, antibody-like in nature, is designed to target TF, consisting of two human factor VII (fVII) targeting domains linked to human immunoglobulin (Ig) G1 Fc as an effector domain. The efficacy of cell-dependent cytotoxicity (IDCC) induced by hI-con1 was evaluated in 5 h chromium release assays. It was demonstrated that hI-con1 elicits strong cytotoxicity against primary USPC cell lines that are resistant to chemotherapy and characterized by overexpression of TF. hI-con1 may represent a promising new therapeutic tool in the treatment of patients with advanced, recurrent, and/or metastatic USPC resistant to standard treatment methods [158].

#### 3.2.4. Chromium Levels in Uterine Fibroids

Research shows that serum Cr levels are higher in patients with uterine fibroids [159].

### 3.3. Selenium

Selenium (Se) plays a crucial role in the human body through its unique activity in preventing cancer occurrence, reducing drug toxicity, regulating thyroid function, and ensuring proper immune system function, thereby playing a significant role in combating diseases, B-lymphocyte proliferation, and enhancing T-cell function. It also serves as a vital component of antioxidants such as glutathione peroxidase and thioredoxin reductase, as well as participating in the functioning of selenoproteins, which perform various functions related to protection against oxidative stress, regulation of redox signaling, thyroid hormone metabolism, and immune system support. This element is present in at least 11 selenoproteins [39,160,161,162].

#### 3.3.1. Selenoproteins

By interacting with the immune system, selenium enhances the activity of immune cells. Selenium is delivered to the human body through food or dietary supplements in two forms: organic (selenomethionine and selenocysteine) and inorganic (selenite and selenate). Both of these forms are converted into selenides, which directly participate in the formation of selenoproteins [163]. Research has also shown that selenium may have antiviral properties. However, there is also a link between selenium deficiency and the association of developing cancer in humans. Due to its potential antiviral activity, it is important to explore the possibility of using selenium as a complementary therapy in the case of cervical cancer. Analysis of tissue samples from cervical cancer tumors and cell lines from this type of cancer, identified with HPV infection, has shown a decrease in the expression of the *selenium-dependent glutathione peroxidase 3* gene. This suggests a potential role of selenoproteins in the development of cervical cancer and HPV infection. Furthermore, the anticancer activity of selenium is often associated with the activity and functions of selenoproteins. Therefore, greater attention should be paid to the role of selenoproteins in the context of cervical cancer. The exact mechanism by which selenium deficiency affects cancer processes is not yet fully understood. However, selenium, as a cofactor of glutathione peroxidase, plays a significant role in preventing the peroxidation of polyunsaturated fatty acids, which leads to a reduced risk of cell membrane damage [164].

#### 3.3.2. Sodium Selenite

In studies on cervical cancer, particular attention has been focused on sodium selenite (Na_2_SeO_3_). This compound serves as an excellent example of a selenium substance with redox properties. Its ability to generate reactive oxygen species is the main reason for its toxicity in cancer cells.

Sodium selenite induces oxidative stress in cells, leading to inhibition of DNA synthesis, DNA damage, and activation of the p53 pathway. Studies have shown that sodium selenite affects the expression of 13 proteins, including those involved in redox balance, apoptosis, signal transduction, mRNA transcription, protein translation, degradation, and translocation. Increased reactive oxygen species production and decreased mitochondrial membrane permeability, along with reduced regulation of antioxidant proteins, suggest that sodium selenite induces apoptosis in HeLa cells through a reactive oxygen species-dependent mitochondrial pathway [164].

Research has shown significant benefits of additional selenium supplementation in the form of sodium selenite for patients with cervical and endometrial cancer, especially in cases of selenium deficiency and when experiencing diarrhea induced by radiotherapy. Analysis of data on disease-free survival and OS suggests that selenium supplementation does not interfere with the beneficial biological effects of radiotherapy, making it an attractive option as a complementary therapy, particularly for patients with marginal levels of this element [165].

#### 3.3.3. Organic Selenium Compounds

Organic selenium compounds can be either natural or synthetic, and their diversity includes differences in chemical structure, cellular penetration, metabolic pathways, and biological effects. Due to their lower toxicity and higher selectivity towards cancer cells compared to sodium selenite, they are considered more promising candidates for anticancer drugs [164].

#### 3.3.4. Nanoparticles of Selenium (SeNPs)

In recent years, special attention has been given to the potential use of selenium nanoparticles (SeNPs) in cancer therapy. The mechanism of action of selenium nanoparticles in chemotherapy (SeNPs) can be explained by the overexpression of antioxidant enzymes, leading to increased generation of reactive oxygen species (ROS). This, in turn, initiates a sequence of events, including the induction of the apoptotic pathway and mitochondrial dysfunction. As a result of this dysfunction, cytochrome C is released, leading to the activation of the caspase cascade. The entire process may lead to cell cycle arrest and DNA fragmentation. Positive results of SeNPs action have been observed on cervical cancer cells (HeLa) [166]. Various modifications of SeNPs, such as the addition of additional factors to their structure, have improved their therapeutic potential, especially in the context of cervical cancer treatment. For instance, adding salicylic acid (SA) to SeNPs can enhance their ability to target cancer cells and penetrate them. Additionally, SA-decorated SeNPs (SA-SeNPs) exhibit low toxicity to healthy cells, as confirmed by experiments on human kidney cells (HK-2). The mechanism of cytotoxic action of SA-SeNPs in cancer cells, such as HeLa cells, involves the induction of apoptosis through caspase-3 activation and PARP degradation. SeNPs also show promising effects as a photothermal therapy (PTT) agent, inducing high temperatures in cells upon irradiation, consequently leading to cell death. Combining PTT therapy with molybdenum diselenide (MoSe_2_) nanocrystals has demonstrated cytotoxic efficacy in HeLa cells, suggesting the potential use of MoSe_2_ nanocrystals as a novel and effective photothermal therapy agent [164]. Additionally, selenium nanoparticles (SeNPs) can be utilized for drug delivery, allowing for a synergistic therapeutic effect in combating cancer. Paclitaxel (PTX) is a well-known anticancer drug that acts by inhibiting the mechanism of mitosis or polymerizing β-tubulin, leading to cell cycle arrest and cell death. Despite its effectiveness, PTX has limitations such as low solubility and a narrow therapeutic index. To improve these properties, PTX has been conjugated with selenium nanoparticles modified with chitosan. Chitosan, due to its structural characteristics such as membrane penetration ability, tissue biocompatibility, and mucoadhesive properties, has been utilized to enhance the solubility and therapeutic efficacy of PTX. This synthetic nanosystem can be perceived as a biocompatible, environmentally friendly, and cost-effective anticancer therapeutic agent developed to combat cancer. Potentially, it could significantly improve human health through cancer diagnosis and treatment. One of the main limitations of selenium nanoparticles is their undesired toxicity to healthy tissues [166].

Silencing genes through short interfering RNA (siRNA) molecules appears to be a promising strategy in cervical cancer therapy. In the realm of drug/gene carriers for anticancer therapy, selenium nanoparticles (SeNPs) are widely utilized.

An active delivery carrier was prepared, with positively charged RGDfC peptide selected for placement on the surfaces of SeNPs to prepare RGDfC-SeNPs gene delivery carrier, as the RGDfC peptide can tightly bind to integrin avb3, which is highly expressed in various types of cancer cells, including HeLa cervical cancer cells. Additionally, positively charged RGDfC-SeNPs interact favorably with siRNA through their electrostatic interaction. HeLa cells showed significant uptake of RGDfC-Se@siRNA through clathrin-mediated endocytosis and exhibited faster siRNA release from RGDfC-SeNPs under acidic conditions. RGDfC-Se@siRNA effectively silenced the *Derlin1* gene in HeLa cells and inhibited proliferation as well as invasion/migration of HeLa cells. Moreover, RGDfC-Se@siRNA was highly effective in suppressing cervical cancer growth in vivo. The study provides promising insights into the design of siRNA carriers for cervical cancer treatment [162,164].

#### 3.3.5. Selenium in Mushrooms

The impact of selenium and *Cordyceps sinensis* mushrooms on potential anticancer properties was investigated. *C. sinensis*, mushrooms used in Chinese medicine, showed promising anticancer activity in in vitro studies. Additionally, seeds from the *Myristica fragrans* Houtt herb, a rich source of selenium, were examined. Supplementation with a water extract from the crushed husk of this herb demonstrated the ability to reduce the incidence of cervical cancer [164].

#### 3.3.6. Selenium as a Marker

The selenium content in tumor tissues post-surgery, benign tumors, and healthy surgical margins has been examined. Lower average selenium levels and GSH-Px activity were observed in the blood serum of patients with tumors and benign tumors compared to healthy women. This suggests that the overall decrease in selenium levels and weakened antioxidant capacity of the body, selenium-dependent, may partially contribute to the development of reproductive system tumors. Following surgery, tissues from patients exhibited higher selenium concentrations in cervical and uterine tumor tissues as well as in benign uterine tumors compared to corresponding healthy tissue margins. This may indicate that the greater accumulation of selenium in these tumor tissues results from the reinforcement of antioxidant defense systems in tumors, which often experience chronic oxidative stress [167].

#### 3.3.7. Selenium as a Serum Marker

It has also been observed that selenium deficiency can result from the cancer itself. Selenium deficiency may be caused by low selenium content in food products, which mainly contributes to human selenium intake [39]. Research has shown significant differences in serum selenium levels in patients with cancer, with the lowest concentrations typically observed in those patients who had distant metastases, experienced multiple recurrences, and had short survival times. Low serum selenium levels may indicate poor nutritional status in patients [168]. The functions of selenium in the body and the role of selenoproteins suggest that this element may play a significant role in the process of carcinogenesis [163]. Analyses regarding the relationship between selenium levels in blood and the risk of endometrial cancer have shown reduced levels of this element in women with endometrial cancer compared to healthy women. Selenium concentration correlates with an increased risk of developing endometrial cancer, so individuals with lower levels of this element should be considered a high-risk group requiring appropriate preventive measures [163,168,169]. There are also other potential mechanisms through which selenium deficiency may contribute to the development of cancer [168].

#### 3.3.8. Selenium as a Urine Marker

There are studies examining the relationship between selenium levels in urine and the risk of cervical cancer. A simple chemical method has been developed to concentrate trace amounts of selenium from large urine samples using small activated carbon filters. Upon irradiation with thermal neutrons, selenium in the samples can be detected through the emission of 77mSe. The results showed a statistically significant increase in selenium excretion in patients with cancer compared to the control group. Additionally, selenium excretion in urine was highest in patients in the intermediate stages of the disease [170].

### 3.4. Boron

Researchers conducted a study to investigate the impact of a binuclear boron–fluoride complex on endometrial cancer. The results revealed that this complex had a strong inhibitory effect on the ECC-1 human endometrial adenocarcinoma cell line. Experimental results demonstrated a significant sensitivity of ECC-1 cells to [L(BF_2_)_2_] compared to other cell lines, as evidenced by an IC_50_ value of 38.36 uM. Researchers have discovered that [L(BF_2_)_2_] significantly impacts ECC-1 cells, specifically by damaging their DNA and initiating a series of processes that ultimately result in apoptosis. This suggests that it could be a potential option for future studies on cancer treatment [171]. A study was conducted in Türkiye to examine the correlation between boron consumption and cervical cytopathology [172]. Women from boron-rich locations had a mean food consumption of 8.41 mg/day of boron, while women from boron-poor regions had a mean dietary intake of 1.26 mg/day of boron (*p* < 0.0001). Women residing in boron-rich regions showed no cytopathological evidence of cervical cancer, but 15 women from boron-poor areas exhibited cytopathological results (*p* < 0.05). The findings indicate that consuming boron in drinking water reduces the occurrence of histological findings associated with cervical cancer. There was no relationship between the clinical findings observed in the cervical smears and the frequency of buccal cell micronuclei. This implies that genetic damage-causing substances equally exposed both research populations. The hypothesis proposed that the interference of B chemistry in the life cycle of HPV could be responsible for this effect. However, researchers found no association between B chemistry and the incidence of oral malignancies caused by HPV [172]. Studies have revealed that serine protease inhibitors have the ability to decrease the immortalizing and transforming effects of the HPV *E7* oncogene [173]. Additionally, studies have shown that plasminogen activator inhibitor-1, a serine protease inhibitor, can reduce the invasive capabilities of cancer cells [173]. Due to the predominance of BA, an inhibitor of serine proteases, in the human body, it was believed that consuming increased quantities of B by drinking water would hinder HPV transformation, hence decreasing the occurrence of cervical cancer [172].

### 3.5. Manganese

Manganese, as a cofactor of SOD, participates in oxidative stress reactions. Studies have shown significantly higher levels of manganese in the tissues of patients with endometrial cancer (EC) compared to other endometrial pathologies. However, logistic regression analysis did not indicate that serum manganese concentration is a risk factor for endometrial cancer.

Diet is the main source of zinc, copper, iron, and manganese intake. General exposure to these metals also occurs through water consumption, air inhalation, and skin contact with products containing these metals. There is also a risk of occupational exposure. Individuals living near or working in mining industries may be exposed to manganese dust or copper fumes [174,175].

#### 3.5.1. MnSOD

Manganese superoxide dismutase (MnSOD) is a significant antioxidant enzyme, serving as the first line of defense against reactive oxygen species (ROS) or free radicals in human cell mitochondria. It catalyzes the conversion of superoxide ions into hydrogen peroxide (H_2_O_2_), which is then converted into water by catalase and glutathione peroxidase [176,177]. MnSOD is encoded by a gene located in the cell nucleus, but its antioxidant function is carried out in the mitochondria [176].

The most common functional polymorphism in the *MnSOD* gene is the substitution of a nucleotide T with C at position -9, resulting in the exchange of valine (GTT) for alanine (GCT). This change affects the enzyme’s localization and transport to mitochondria. It has been shown that the CT/CC rs4880 *MnSOD* genotype is associated with a reduced risk of developing CIN1 (cervical intraepithelial neoplasia), especially in individuals with higher levels of β-carotene and γ-tocopherol. Similar results were also observed for lycopene and α-tocopherol, which were associated with lower risks of CIN2/3. In mitochondria, higher expression of *MnSOD* may lead to increased levels of H_2_O_2_, which are produced by the dismutation of superoxide anions, and this may induce toxicity if glutathione peroxidase activity or antioxidant levels are low. However, when antioxidant levels are adequate, the increased rate of superoxide anion quenching by the C genotype may be beneficial. In this study, it was observed that women with the C genotype and higher antioxidant status appear to be protected against cervical carcinogenesis. Conversely, women with the C allele rs4880 *MnSOD* and low antioxidant status have a higher risk of cervical carcinogenesis than those with the T allele [178].

Single Nucleotide Polymorphisms (SNPs) in the *MnSOD* gene were simultaneously genotyped using the multiplex SNaPshot^®^ system, which is used to investigate Val-9Ala MTS and Ile58Thr exon-3 on two templates in one reaction. The study emphasizes the importance of advanced genotyping techniques, such as the Multiplex SNaPshot^®^ system, in understanding the genetic relationships with complex diseases, such as cancer. It was found that the relationships between genotypes in patients with cervical and breast cancer and healthy women did not reach statistical significance. The Multiplex SNaPshot^®^ system was effectively used to genotype these two significant SNPs in the *MnSOD* gene in one high-throughput SNaPshot^®^ reaction [176].

#### 3.5.2. Mn in Treatment

Radiation therapy (RT) has been playing a crucial role in cancer treatment for many years. However, the abnormal conditions within the tumor microenvironment, such as hypoxia, acidosis, and dense extracellular matrix, can lead to resistance to radiation therapy. To counteract this issue, serum albumin, known as BSA, has been utilized as a carrier to introduce Bi_2_S_3_ and MnO_2_ nanoparticles, creating nanocomposites labeled as BSA-Bi_2_S_3_-MnO_2_ through a biomineralization process. The Bi_2_S_3_ nanoparticles enhanced the efficacy of radiation therapy, while the MnO_2_ nanoparticles catalyzed the decomposition of endogenous H_2_O_2_, generating additional oxygen, thereby increasing the effectiveness of radiation therapy. Additionally, the BSA-Bi_2_S_3_-MnO_2_ nanocomposites also exhibited a photothermal effect, which could improve the tumor microenvironment with hypoxia, further enhancing the efficacy of radiation therapy. Importantly, long-term toxicity of the BSA-Bi_2_S_3_-MnO_2_ nanocomposites was not observed after intravenous administration [178].

The microenvironment of cervical cancer is known to limit the effectiveness of immunotherapy due to its immunosuppressive effects. To overcome these limitations, PEGylated calcium sulfide nanoparticles doped with manganese (MCSP) were investigated to stimulate the anti-tumor immune response through metalloimmunotherapy. Dual activation of pyroptosis and the STING pathway was employed to enhance therapy efficacy. Mitochondrial disturbances induced by H_2_S further increased the release of mitochondrial DNA (mtDNA), strengthening the activation effect of Mn^2+^ on the cGAS-STING signaling axis, and thus activating immunosuppressive dendritic cells. The combination of MCSP nanoparticles with PD-1 immunotherapy demonstrated synergistic anti-tumor effects, effectively inhibiting tumor growth [179].

### 3.6. Cobalt

Cobalt is a trace element that plays a crucial role in maintaining the internal balance of the human body. It is a component of hydroxycobalamin (vitamin B12), which is involved in the formation of red blood cells. Its deficiency can lead to microcytic and macrocytic anemia. In serum, cobalt in the form of Co^2+^ ions is bound to albumin and then becomes a component of vitamin B12. The combination of cobalt with this vitamin is crucial for the proper functioning of the body. Cobalamin acts as a cofactor for two enzymes: methionine synthase and methylmalonyl-CoA mutase. The use of cobalt in industry is associated with the risk of occupational diseases resulting from inhaling its compounds. There are many ways to monitor exposure to cobalt, including measuring its concentration in blood (cobaltemia) and urine (cobalturia). In cells, cobalt occurs as a divalent or trivalent ion, binding to other molecules.

This leads to the formation of oxides, chlorides, or other cobalt compounds. Due to its similarity to iron (II) ions, cobalt can replace Fe^2+^ in the heme ring, resulting in the formation of the reduced form of the protein. This leads to hypoxia, resulting in tissue oxygen deficiency and activation of the *erythropoietin* gene. It is known that cobalt is a strong initiator of oxidative stress in cells, which alters gene function and may increase the risk of cancer. Pure cobalt and soluble cobalt salts (II) can be classified as probably carcinogenic to humans. On the other hand, cobalt oxide (II,III), cobalt sulfide (II), and other cobalt compounds (II) are classified in Group 3 and are not considered carcinogenic. It is worth noting that CoCl_2_ induces hypoxia, leading to inhibition of ovarian cancer cell proliferation [140].

#### 3.6.1. Treatment

Treatment for advanced cervical cancer according to the FIGO classification typically involves chemoradiotherapy, combining chemotherapy and radiotherapy, along with brachytherapy. Following surgical procedures, radiotherapy combined with a drug called cisplatin can also be used to treat advanced cases of the disease. Radiotherapy can be specifically divided into high-dose rate brachytherapy (HDR), which is a more intense form of treatment. Various radioisotopes are utilized in radiotherapy, such as Ir-192 and Co-60, with Ir-192 being more commonly used. Co-60 may be more effective in treating tumors of lower stage and smaller than 2 cm in diameter, which additionally makes it more economically viable. Studies have shown that three cobalt (II) complex compounds containing 8-methoxyquinolines (MQL) such as [Co(MQL)_2_Cl_2_] (CoCl_2_), [Co(MQL)_2_Br_2_] (CoBr), and [Co(MQL)_2_I_2_] (CoI), may have greater anti-proliferative efficacy than cisplatin in treating ovarian cancers resistant to cisplatin. In the case of cervical cancer cells (HeLa) exposed to complex 2 [CoIII(4,4′-Me2-bpy)3](PF6)3, only high concentrations of this complex resulted in the arrest of HeLa cells in the S phase of the cell cycle. To confirm this observation, levels of proteins regulating the cell cycle from the G1 to S phase were analyzed. Increased accumulation of cyclin A and E2F was observed, suggesting that complex 2 may inhibit the proliferation of cancer cells by arresting them in the S phase [140].

#### 3.6.2. The Cobalt (III) Complex

The complex cobalt (III) with a Schiff base, named M3, was synthesized and subjected to detailed characterization using single-crystal X-ray analysis. Its cytotoxic effects were also investigated across various cell lines including HeLa, LoVo, A549, A549/cis, and the normal cell line LO2, using MTT assays. It was found that complex M3 inhibits cell proliferation by blocking DNA synthesis, leading to the disruption of nuclear division in the HeLa cell line. Additionally, Western blot analysis revealed that M3 significantly reduced the expression levels of two proteins, c-Myc and KLF5, while also activating various signaling pathways such as ER stress, apoptosis, cell cycle, and DNA damage in HeLa cells. It is worth noting that M3 had no impact on proteasome activity, suggesting a different mechanism of action for this compound [180].

#### 3.6.3. Cobalt-60

When comparing the biological effectiveness of doses (BED) to target volumes (CTV) and organs at risk (OAR) in women with cervical cancer treated with high-dose rate brachytherapy (HDR-BT) using either iridium-192 (192 Ir) or cobalt-60 (60 Co), various factors were considered. These factors included repair during fractions, tumor growth rate, differences in radiation sensitivity related to hypoxia, and radiation biological effectiveness (RBE). In comparing doses to CTV and OAR, it was found that increasing the dose by approximately 4% is feasible and safe while minimizing damage to healthy tissues, regardless of the radiation source used, whether 60 Co or 192 Ir [181].

In studies focusing on cervical cancer using a dynamic fractionation regimen with cobalt-60 radiation, coupled with normobaric oxygen breathing, a reduced response of healthy tissues was observed compared to the standard dosing scheme. Exophytic tumors, characterized by better vascularity, appear to respond more favorably to treatment than endophytic tumors, which are less vascularized [182].

Due to its long half-life, cobalt-60 offers logistical and economic savings. The reduced need for source changes allows for easier access to brachytherapy, which is crucial for providing optimal care for cervical cancer patients. Brachytherapy using HDR Co-60 can be particularly beneficial in low- and middle-income countries where resources are limited. Studies have shown that patients treated with HDR Co-60 brachytherapy achieved comparable survival outcomes and therapy tolerance compared to HDR Ir-192 brachytherapy [183].

#### 3.6.4. Co-Ferritin

Cytotoxicity, intracellular uptake, and metabolomic profiles of HeLa and HaCaT cell lines were studied following exposure to cobalt ferrite nanoparticles (CoFe_2_O_4_ NPs). It was found that the level of fumarate, an oncometabolite, was decreased in HeLa cells after nanoparticle treatment, which may influence the inhibition of carcinogenesis processes. Fumarate is known to inhibit prolyl hydroxylase, leading to the stabilization of HIF1α, a key regulator of carcinogenesis that promotes tumor growth and development. The results suggest that HeLa cells treated with nanoparticles exhibited reduced concentrations of cell proliferation- and tumor growth-related metabolites. Ultimately, it was observed that therapy with these nanoparticles may disrupt cellular metabolism [184].

#### 3.6.5. Uterine Fibroids Development

Elevated levels of cobalt were observed in the urine of individuals with uterine fibroids. Increased exposure to cadmium, lead, and/or cobalt may impact fibroid development by interacting with estrogen receptors. This can lead to a bidirectional mechanism where increased exposure initiates fibroid growth, followed by increased permeability and retention of metals in fibroid tissue. This makes fibroids act as reservoirs for these metals, increasing their levels in blood and urine and systemic exposure to them. Alternatively, fibroids may simply serve as storage for metals due to altered vascular structure within fibroids. In this scenario, they are not a direct cause of increased metal exposure but can still influence their prolonged presence in the body [185].

#### 3.6.6. Summary

A review of studies suggests that cobalt-containing compounds may be factors that increase the risk of cancer development and accelerate its progression. However, cobalt ions themselves do not appear to directly cause such risk. Cobalt salts may disrupt DNA repair processes, contributing to the formation of mutagenic factors and, consequently, to the development of tumors. Additionally, the binding of HIF-1α by cobalt may mimic hypoxic conditions, which also promotes cancer development. Despite these unfavorable properties, due to its specific physical characteristics, cobalt may be useful in the radiotherapy of gynecological tumors, especially in countries with limited financial resources for cancer therapy (Table 2) [140].

### 3.7. Cobalt in Cervical Cancer

Treatment for advanced cervical cancer, starting from stage IB2, relies on chemoradiotherapy with brachytherapy. Post-surgery, if necessary, radiotherapy with cisplatin is administered. For gynecologic tumors such as bladder and rectum, radiotherapy is the main therapeutic method. In radiotherapy for cervical cancer, isotopes such as Ir-192 and Co-60 are mainly used, although iridium is the more commonly used isotope. Studies suggest that differences between Ir-192 and Co-60 in terms of efficacy and toxicity in brachytherapy may be minimal. There is speculation that cobalt-60 may be more effective for tumors at lower stages or smaller diameters, such as those less than 2 cm. The cost of therapy is an important factor in choosing the isotope for gynecologic cancer treatment, where cobalt is more economically favorable due to its longer half-life. This necessitates more frequent iridium source exchanges, which incur higher costs. Additionally, cobalt is being explored as a potential anticancer drug due to its lower toxicity and findings suggesting that some cobalt complexes may exhibit greater antiproliferative activity than cisplatin, particularly in cisplatin-resistant ovarian tumors. Furthermore, there is evidence suggesting that three cobalt (II) complexes with 8-methoxyquinoline (MQL)—[Co(MQL)_2_Cl_2_] (CoCl_2_), [Co(MQL)_2_Br_2_] (CoBr), and [Co(MQL)_2_I_2_] (CoI)—may potentially be more effective in inhibiting the growth of cisplatin-resistant ovarian tumors than cisplatin alone. Studies on cervical cancer cells (HeLa) treated with the complex 2 [CoIII(4,4′-Me2-bpy)3](PF6)3 showed that only high concentrations of this complex were able to halt the growth of HeLa cancer cells in the S phase [140].

### 3.8. Iodine

There is no evidence of iodine influence in endometrial cancer. Staddel in 1976 suggested that geographic variations in breast, endometrial, and ovarian cancer rates seem to show an inverse relationship with dietary iodine intake, but data from sub-Saharan African countries opposed the hypothesis [186,187]. The incidence of breast, endometrial, and ovarian cancer is lower than in high-income countries [1,187]. From an endocrinological perspective, low dietary iodine intake could lead to heightened gonadotrophin stimulation, potentially resulting in a hyperestrogenic state characterized by elevated levels of estrone and estradiol and a decreased estriol to estrone plus estradiol ratio [186].

### 3.9. Fluoride

Fluoride is widely recognized for its role in dental health, particularly in preventing dental caries by enhancing enamel resistance to acid demineralization. However, its biological effects beyond dental applications are complex and less understood. In biological systems, fluoride impacts various cellular processes, including enzyme activity, signal transduction, and oxidative stress, making it a subject of interest in cancer research.

Fluoride influences oxidative stress pathways, which are pivotal in cancer development, by increasing oxidative stress that can lead to DNA damage and promote carcinogenesis [188]. It affects key antioxidant defense enzymes, including SOD and catalase, altering their activities and thereby impacting cellular protection mechanisms against oxidative damage [188,189]. Furthermore, fluoride can modify cellular signaling pathways associated with cell proliferation and apoptosis, thus influencing tumor growth and progression [188,189].

Recent studies have identified several oxidative stress biomarkers affected by fluoride, including malondialdehyde (MDA), a lipid peroxidation product, and 8-hydroxy-2-deoxyguanosine (8-OHdG), an indicator of DNA base guanine oxidation [190,191]. Advanced oxidation protein products (AOPP) and advanced glycation end-products (AGEs) are also recognized as markers of protein and carbohydrate oxidative stress, respectively. Research has shown that fluoride exposure significantly increases 8-OHdG levels in various tissues, such as testis, plasma, SH-SY5Y cells, and TCMK-1 cells [192,193,194,195]. Elevated AOPP levels have been found in rat serum and adult plasma in fluorosis areas, while AGEs are increased in brains and SH-SY5Y cells exposed to fluoride [196,197]. These findings underscore fluoride’s substantial impact on oxidative stress across lipids, genes, proteins, and carbohydrates, elucidating its potential role in cancer biology.

A study by Zhou et al. on sodium fluoride (NaF) and female fertility revealed that NaF increased estrogen receptor alpha protein (ERα) expression, particularly at high concentrations (100 mg/L) [198]. This increase in ERα could lead to decreased endometrial receptivity and impaired embryo implantation. Both ERα and progesterone receptor proteins were upregulated in high fluoride groups, suggesting that fluoride’s lower ionic mobility might contribute to these hormonal changes. These hormones are critical for oocyte development and pre-implantation of the uterine walls, highlighting the need for further research into fluoride’s impact on reproductive health.

Recent studies have highlighted fluoride’s potential in treating endometrial carcinoma (EC). A notable development is the discovery of a novel binuclear boron–fluoride complex, [L(BF_2_)_2_], which exhibits promising anticancer properties [171]. This compound opens new avenues for developing chemotherapeutic agents.

A study utilizing two cell lines—adenocarcinoma of the human endometrial cell line ECC-1 as the target and normal prostate epithelium cell line PNT1-A as a control—examined the effects of [L(BF_2_)_2_] and cisplatin (used as a positive control). The study aimed to assess the apoptotic, cytotoxic, and genotoxic effects of [L(BF_2_)_2_] through various assays, including MTT, ELISA, DNA laddering, Comet assay, and acridine orange staining. Additionally, total antioxidant status (TAS), total oxidant status (TOS), and ferric reducing antioxidant power (FRAP) were measured in the ECC-1 cell line.

ECC-1 cells exhibited high sensitivity to [L(BF_2_)_2_] with an IC_50_ value of 38.36 µM, slightly different from the control PNT1-A cells. Initial exposure to 2, 5, and 10 µM of [L(BF_2_)_2_] did not affect ECC-1 cell growth compared to controls. However, at 25 µM, [L(BF_2_)_2_] significantly reduced cell proliferation in both cell lines, similar to the effect of cisplatin, though less pronounced in PNT1-A cells.

The results prompted further testing, showing that [L(BF_2_)_2_] increased oxidative stress and caused irreparable DNA damage, leading to cell apoptosis. These findings suggest [L(BF_2_)_2_] has potential as a chemotherapeutic agent for endometrial cancer, warranting additional studies to explore its clinical properties and mechanisms in vivo.

### 3.10. Zinc and Copper

Zinc and copper are essential for maintaining the health of the reproductive system due to their roles as antioxidant enzymes [14]. Zinc is crucial for DNA synthesis, repair, and cellular proliferation, and disturbances in zinc homeostasis have been linked to cancer progression. In uterine cancer, research is focusing on zinc’s role in tumor growth and patient prognosis. Most zinc in the human body is stored in skeletal muscle and bones, with a small fraction circulating in the bloodstream bound to proteins [199]. The regulation of intracellular zinc levels is redox-sensitive, and zinc enhances the activity of the nuclear transcription factor Nrf2, which regulates detoxification mechanisms [199]. Additionally, zinc serves as a cofactor for enzymes such as Cu/Zn-superoxide dismutases, which are essential for antioxidant defense [200].

Copper exists in two oxidation states, Cu(I) and Cu(II), making it redox-active. In the bloodstream, copper is mainly bound to proteins such as ceruloplasmin, albumin, and alpha-2-macroglobulin, with a portion acting as a cofactor for extracellular superoxide dismutase (SOD3) [200]. Both zinc and copper are critical to antioxidant and inflammatory processes and play significant roles in oncogenesis and malignant transformation of cells.

Atakul et al. evaluated serum concentrations of copper (Cu) and zinc (Zn) in relation to the metabolic profile and clinicopathologic features of endometrial cancer patients [123]. Clinicopathologic features, metabolic profiles, and serum Cu and Zn levels were assessed. Women with endometrial cancer had lower Cu and Zn levels compared to controls. The Cu/Zn ratio was higher in controls compared to cancer patients. Zn levels showed a positive correlation with parity. In cancer patients, both Cu and Zn levels positively correlated with age. Those with myometrial invasion > 1/2 had lower Cu levels than those with myometrial invasion < 1/2. The study suggests that altered serum Cu and Zn levels are associated with endometrial cancer in Turkish patients.

Kluza et al. investigated the relationship between serum copper (Cu) and zinc (Zn) levels and the incidence of endometrial cancer [201]. They involved 306 participants, equally divided into a test group (patients with endometrial cancer) and a control group, matched by age. The test group had significantly lower levels of Cu and Zn compared to the control group. There was a statistically significant association between lower Cu and Zn levels and a higher incidence of endometrial cancer.

Patients with the lowest Cu levels had an 8.54 times higher risk of developing endometrial cancer. Patients with the lowest Zn levels had a 15 times higher risk of developing endometrial cancer. The study suggests that lower serum levels of Cu and Zn are associated with an increased risk of endometrial cancer.

### 3.11. Arsenic

Arsenic trioxide (ATO) has demonstrated its significant clinical efficacy in treating promyelocytic leukemia patients [202]. ATO exhibits anti-tumor activity against various solid tumors, including osteosarcoma, hepatocellular carcinoma, breast cancer, and lung cancer [203]. Recent studies have also suggested ATO’s potential in treating endometrial cancer. Zhou et al. (2007) found that ATO inhibited proliferation, reduced telomerase activity, and induced apoptosis in four endometrial cancer cell lines [204]. Additionally, ATO has been reported the anti-estrogenic activity in endometrial cancer cells through the mitogen-activated protein kinase (MAPK) signaling pathway [205]. Cai et al. reported the first ATO treatment of platinum-resistant recurrent endometrial cancer [203].

### 3.12. Cadmium

Cadmium (Cd) is a rare element that has been classified as a human carcinogen by the World Health Organization (WHO) [206]. Research showed that there is a positive correlation between Cd exposure and increased number of endometrial cancer onset. A case-control study conducted on a midwestern population of the United States revealed that there is a 22% elevated risk of endometrial cancer occurrence due to Cd exposure [207]. A study conducted on 110 females in Szczecin, Poland also confirmed a higher rate of endometrial cancer onset in a group of patients with elevated Cd blood concentration (OR = 5.25; 95% CI 1.56–17.72) [208]. Humans may be exposed to Cd by smoking or by consuming contaminated food. During such exposure, Cd may interact and negatively influence cells by multiple pathways. Cd leads to not only increased oxidative stress but also can contribute to inhibiting the damaged DNA repair. Templeton et al. showed that oxidative damage of thiol groups in cellular component peptides caused by Cd may result in denaturation and destruction via the proteasomes [209]. Moreover, Cd might suppress the process of apoptosis. It has been proved that Cd can decelerate the activity of caspase-3, caspase-8, and caspase-9, which are crucial for chromatin condensation and fragmentation of DNA and consequently for proper cell apoptosis. Cd can also affect mitochondria and inhibit the emission of pro-apoptotic cytochrome C from them [209,210]. Furthermore, there is a hypothesis that Cd operates as a powerful metallohormone that might mimic the role of steroid hormones. By simulating the function of estrogen, Cd may regulate the growth of epithelium and potentially induce endometrial cancer development [208,211]. Razumowa et al. showed that dietary intake of Cd more than 13.1 μg was related to a significant decrease in OS of endometrial cancer patients (HR = 0.956, 95% CI = 0.914–1.001, *p* = 0.05) (Table 3) [212].

## 4. Vitamins

### 4.1. Vitamin A

Vitamin A is a soluble in oil compound essential for the development of healthy skin and hair [213]. There is few information about the correlation between vitamin A and uterine cancer. However, there are some reports presenting the potential use of retinoids in the therapy of endometrial malignancies. Vitamin A is known for estrogen metabolism-modulating factors. Due to managing the function of 17β-Hydroxysteroid dehydrogenase type 2 (17-HSD) and stimulating its activity, vitamin A might modify the serum level of estrogen. By converting estrogens into lower-activity forms, 17-HSD type 2 enzyme may limit the amount of active sex steroids that can enter the bloodstream. By reducing the level of estrogen which excess is one of the endometrial cancer development risk factors, vitamin A may decrease the risk of tumor onset or its progression [214,215,216]. Moreover, Cheng et al. reported about possible inhibition features of retinoids. The use of retinoid acids significantly reduces the growth of the Ishikawa endometrial cancer cell line probably by inhibiting the cell cycle at the G1 phase. Results of this study also showed that retinoids may inhibit the cell cycle of endometrial cancer cells by modulating the function of retinoic acid receptor alpha (RAR-α). Results showed that retinoid acids reduce the expression of RAR-α and decreased activity of RAR-α leads to greater anti-apoptotic BCL2 protein and proliferating cell nuclear antigen (PCNA) which are crucial for proper endometrial cell differentiation and growth [217,218]. Furthermore, Pelucchi et al. presented a correlation between retinoid intake and decreased risk of endometrial cancer onset. When beta-carotene consumption was compared between the top and lowest quartiles, the ORs were significantly lower (OR = 0.69, 95% CI, 0.48–0.99). In their case-control study, it has been suggested that the antioxidative properties of retinoids may protect DNA from oxidative damage by triggering apoptosis in altered cells and modulating the immune system response [219].

### 4.2. Vitamin B Group

The B-complex vitamins are a set of eight water-soluble vitamins that can be sourced from meat, green vegetables, and dairy products. The group of vitamins is crucial for metabolism functioning, neurotransmitter synthesis, and even for proper expression of nucleic acids [220]. Research showed that folate intake may be associated with decreased risk of endometrial cancer occurrence. There are reports about increased endometrial cancer onset in a group of women with folate deficiency. Insufficient amounts of folic acid may diminish the availability of 5-methyltetrahydrofolate, which is required for the remethylation of homocysteine to methionine. Such a situation might lead to DNA hypomethylation, defects in precursors of DNA, uracil misincorporation, and chromosome damages which all can contribute to DNA repairing abnormalities and endometrial cancer development [221,222,223]. However, there are also studies that presented a positive correlation between increased folate intake and elevated risk of endometrial cancer development. Du et al. suggested that increased folate levels can additionally enhance the synthesis of DNA in rapidly proliferating cells, and accelerate the progression of existing tumors [223,224]. Furthermore, folate can potentially affect immune system activity. It has been discovered that supplementation of more than 400 μg/d might impair the function of NK cells [223]. There is poor information about the possible association between thiamine, riboflavin, niacin, pyridoxine or cobalamin and the endometrial cancer onset. However, Sundravel et al. presented results of the influence of riboflavin and niacin intake on the progression of endometrial cancer in animal models. In the group of rats treated with the combination of riboflavin, niacin, and ascorbic acid, the activity of enzymes such as aldolase, hexokinase, and phosphoglucoisomerase was significantly inhibited. Aldolase, hexokinase, and phosphoglucoisomerase, which are glycolytic enzymes, are crucial for the catabolism of glucose and for supplying energy to tumors. Without the catabolic feature of the enzymes, rapid growth and progression of endometrial cancer might be suppressed. Results showed that a combined intake of niacin, riboflavin, and ascorbic acid might be a potential therapeutic strategy for endometrial carcinomas [225].

### 4.3. Vitamin C

Vitamin C, also known as L-ascorbic acid, plays a significant role in human physiological processes including hormone production, immunological modulation, and antioxidative activity [226,227]. Moreover, research shows that vitamin C supplementation might reduce the risk of endometrial cancer onset. Chen et al. described a correlation between vitamin C intake and improved transcriptional activity. Numerous genes involved in angiogenesis, glucose and iron absorption, and cell growth are transcriptionally induced by hypoxia-inducible factor 1α (HIF-1α) [228,229]. Research also shows that vitamin C presents a potential role in the regulation of HIF-1α activity and its supplementation might reduce the risk of endometrial cancer development. There is a statistical significance between HIF-1α hyperactivity and low vitamin C concentrations (*p* = 0.007) [230]. Due to its antioxidant properties, vitamin C may regulate the action of HIF-1α, the activity of which remains in correlation with oxygen concentration [228,229]. In people with higher vitamin C serum levels, the activity of HIF-1α was remarkably lower than in the group with vitamin C deficiency [231]. Markowska et al. pointed at the association between more aggressive types of endometrial cancers and increased activity of HIF-1α [230]. Furthermore, vitamin C also regulates the metabolism of metal ions such as copper or iron. By modulating multiple iron-dependent enzymatic reactions, vitamin C can affect the DNA synthesis and protect from the potential DNA damage [228]. Kim et al. presented that the activity of vitamin C might regulate the expression of p53, which is one of the regulatory proteins with cancerogenesis suppression features [232]. The p53 dysregulation plays a significant role in the development of different cancers. Different studies show that vitamin C intake may regulate the p53 activity by promoting the phosphorylation of p53 and in turn increase the amount of ROS. Increased ROS generation might potentially damage cancer cells and lead to tumor growth inhibition [233,234].

### 4.4. Vitamin D

Vitamin D is a crucial compound for the proper function of the skeletal, immunological and neurological systems that also presents many other benefits for human health [235,236]. Although Vitamin D supplementation may be beneficial for different diseases, the potentially positive influence on reducing the risk of uterine cancer onset is unclear. Systemic reviews and prospective cohort analysis present no considerable evidence of the link between vitamin D intake and the development of endometrial cancer [36,237,238]. However, Deuster et al. suggested the potential anti-cancer activity of vitamin D due to its influence on maintaining proper weight. In animal models, vitamin D supplementation demonstrated inhibition of the growth of premalignant endometrial lesions [239]. Research shows that vitamin D may be used in endometrial cancer therapy. A study conducted on HEC-1A cells presented that a combination of vitamin D and perifosine significantly reduced cancer cell proliferation compared to the control group [240]. It has been demonstrated that vitamin D inhibits proliferation through several processes, such as inhibition of the cell cycle, differentiation promotion, and apoptosis [241]. The combined use of vitamin D with chemotherapeutics such as perifosine which inhibits AKT activation responsible for the regulation of cell growth might lead to higher inhibition of cancer progression [240]. Moreover, Kuittinen et al. presented a promising combined therapy for endometrial cancer which consists of paclitaxel, carboplatin, and vitamin D. Results showed that the combination of two chemotherapeutics with vitamin D contributes to higher cell growth inhibition than using single chemotherapy [242].

### 4.5. Vitamin E

Vitamin E is a lipid-soluble vitamin that plays an important role in the proper function of the immune, cardiovascular, or neurological systems [243]. Research showed that vitamin E, in addition to its well-known positive functions on human physiology, may also decrease the risk of endometrial cancer onset. Xu et al. presented that in a group of women receiving vitamin E supplements, the occurrence of endometrial cancer was significantly lower (OR = 0.8, 95% Cl, 0.5–1.1) [244]. The possible cause of such anti-cancer activity of vitamin E may be its antioxidative properties. Vitamin E gathers peroxyl radicals and prevents the oxidation of polyunsaturated fatty acids (PUFAs). Substituted for lipid hydroperoxide, one of the forms of vitamin E (alpha-tocopherol) reacts with peroxyl radicals, preventing continued oxidation of PUFAs [244,245,246]. Studies showed that supplementation of 5mg/1000 kcal of vitamin E might reduce the risk of endometrial cancer development [231].

However, compared to previously mentioned evidence, Markowska et al. reported a correlation between lower expression of alpha-tocopherol transfer protein (αTTP) and a less aggressive course of endometrial cancer. αTTP promotes vitamin E transport to cells and affects ROS generation. Results showed that in a group of women with endometrial cancer, reduced αTTP expression was associated with lower FIGO stage I/II and a significantly higher 5-year survival rate (*p* = 0.014 and *p* = 0.041). Cancer cells may probably protect themselves against oxidative stress by impairing the expression of αTTP, which can explain this correlation [247,248]. Furthermore, research showed that the use of suppositories containing vitamin E may decrease the onset of cellular atypia and therefore reduce the risk of cancer onset. Wierzbicka et al. reported that the application of suppositories with vitamin E leads to mucosal regeneration, inhibition of inflammatory processes, and improved differentiation of cells [249].

## 5. Other Elements

### Titanium

A magnetic core–shell nanocarrier made of titanium carbide was used to deliver targeted chemotherapy. The magnetic control of the nanocarrier to release the chemical cisplatin significantly suppressed tumor growth in a cervix xenograft model. When administered together with medications, it has synergistic therapeutic effectiveness (*p* < 0.001) [250]. Another treatment employs titanium coupled with blue light and C-doped TiO_2_ nanoparticles [251]. Ultraviolet-visible light spectroscopy revealed that adding carbon (C) to titanium dioxide (TiO_2_) results in an increase in light absorption in the visible range. This is due to a reduction in the energy gap between the valence and conduction bands. The in vitro photo-cytotoxic activity of C-TiO_2_ was examined against HeLa cells. The absence of dark cytotoxicity demonstrates C-TiO_2_’s favorable biocompatibility. On the other hand, the presence of blue light significantly lowered HeLa cells’ survival. The exposure of the cells to illumination for 15 min at a power of 120 W resulted in a 30% decline in their viability. Additionally, when HeLa cells were preincubated with C-TiO_2_, their viability plummeted by 60%. Furthermore, it was verified that exposure to blue light led to the production of reactive oxygen species both in laboratory conditions (in vitro) and inside cells (intracellularly), with the assistance of a C-TiO_2_ catalyst. The primary factor responsible for the death of HeLa cells was oxidative stress induced by C-TiO_2_/blue light. Fluorescent tagging revealed distinct morphological alterations in treated HeLa cells after the C-TiO_2_/blue light treatment. Contrary to the effects of blue light illumination, which resulted in the formation of abnormally shaped cells with enlarged dead areas, swollen cytoplasm, and membrane protrusions, the combination of C-TiO_2_ with blue light induces regulated cell death, leading to a more favorable outcome in localized anticancer treatment. However, the implementation of this therapy in clinical applications may pose challenges due to the large bandgap energy (3.2 eV) and the requirement to use UV light for photostimulation. UV radiation cannot penetrate deeply into human tissues and is also hazardous. As a result, TiO_2_-based phototherapy is only effective for treating tumors that are located near the surface of the body.

## 6. Conclusions

Nutritional status of the patients undoubtedly plays a crucial role in the onset and progression of endometrial cancer. Calcium supplementation seems to be a reasonable way to potentially reduce the risk of endometrial cancer onset; however, there are studies that provide contradictory results to the above hypothesis. In addition to calcium, phosphorus, selenium, and zinc intake were also reported to inversely correlate with endometrial carcinogenesis as deficiencies of the abovementioned elements might lead to the onset of endometrial cancers. Conversely, it was observed that excessive bioaccumulation of such elements as iron, copper, or cadmium might positively correlate with endometrial cancer. Nevertheless, many molecular mechanisms of action of the reviewed elements are still unknown and, as presented in our paper, a lot of data are still contradictory and require further evaluation. In addition, the nutritional status of patients constitutes only a component of a vast number of variables that stimulate carcinogenesis; therefore, genetic, individual, and environmental factors should always be taken into consideration. Thus, further research with bigger cohorts and a greater number of elements is crucial to unify the results of the so-far conducted studies.

## Figures and Tables

**Table 1:** Association between micronutrients and endometrial cancer.

Micronutrient	Result	References
Calcium	Estrogen increases intracellular calcium, triggering cellular responses that promote cancer development and progression. Excess Ca in mitochondria generates ROS, facilitating tumor growth. Estrogen also increases proteins like ERRγ and S100A4, which promote tumor invasion. Ca channel blockers statins can help reduce cancer risk and improve survival. Inhibition of calcium channels, such as CACNA1D and TRPV4, can reduce cell survival and migration, enhance apoptosis, and prevent metastasis. High *TRPM4* expression correlates with cancer growth and estrogen response, while *TRPV2* overexpression increases the effectiveness of chemotherapy drugs like cisplatin. Dietary calcium’s impact on endometrial cancer risk is inconclusive, with studies showing both potential protective and positive correlations.	[15,18,19,20,24,25,26,30,36]
Phosphorus	P plays a protective role in endometrial cancer. The NHANES survey (2007–2016) found a significant inverse association between P intake and endometrial cancer risk. Endometrial cancer survivors often have lower P intake than healthy controls. Elevated serum P levels are common in cancer patients, and historical studies have shown cancerous tissues selectively absorb P. Radioactive phosphorus-32 (32P) has been used effectively in the adjuvant therapy of ovarian and endometrial cancers, reducing tumor recurrence and improving survival rates. Black phosphorus nanosheets are being explored as a promising nanocarrier for targeted cancer therapies.	[38,42,43,47,55]
Sodium	Sodium butyrate (NaBu) suppresses tumors by inducing ROS and DNA damage, promoting ferroptosis, and enhancing chemotherapy efficacy. Voltage-gated sodium channels (Nav1.7) are highly expressed in cancer tissues, associated with larger tumors and lower survival rates; blocking Nav1.7 reduces invasion and promotes apoptosis. Epithelial sodium channel genes (*SCNN1A*, *SCNN1B*, *SCNN1G*) are less expressed in tumors, with higher expression linked to better survival and lower tumor grade.	[56,73,76]
Potassium	K intake shows potential inverse correlations with endometrial cancer risk. Voltage-gated potassium channels, including Eag1 (KCNH1) influence cell migration and AKT pathway activation. Inhibition of these channels suppresses metastasis and affects vimentin-related pathways, impacting cancer progression. K channels regulate cell cycle.	[38,83,87,89,90,102]
Magnesium	Mg is critical in endometrial cancer due to its role through MAGT1 in facilitating cell cycle progression and proliferation, especially in HPV-positive cervical cancer cells. Mg valproate therapy up-regulates genes involved in pathways like MAPK signaling and apoptosis, enhancing histone acetylation and activating tumor suppressor genes. Mg supplementation during cisplatin chemotherapy protects kidney function by mitigating nephrotoxicity.	[108,109,110,111]
Zinc	Zn stabilizes DNA and RNA, supports DNA integrity via zinc-dependent proteins, and impacts tumor suppressors like p53, with depletion potentially increasing cancer risk. Zinc’s role in promoting cell growth and membrane integrity suggests cancer cells may uptake Zn for growth and survival. Low Zn levels have been associated with higher risks of endometrial and cervical cancers, and Zn supplements have been shown to mitigate serum deficiencies and may influence HPV infection rates. Zinc-related proteins like RIZ1 impact tumor progression and prognosis in cervical cancer by regulating apoptosis and DNA damage. The zinc finger transcription factors ZEB1 and ZEB2 contribute to EMT, promoting tumor invasion and metastasis across various cancers.	[117,119,121,122,124,125,128,131,136,137,138,139]
Iron	Fe is essential for enzyme actions like catalase and cytochromes, and proteins like hemoglobin. It also generates harmful free radicals, influencing oxidative processes. Heme Fe, prevalent in red meat, increases oxidative stress and DNA damage. High heme iron intake may associate with higher risks of endometrial cancer due to its pro-oxidative and inflammatory effects. In fibroids, Fe levels are lower compared to normal and cancerous tissues. Intravenous Fe administration can rapidly treat anemia in cervical cancer patients undergoing chemoradiotherapy, improving red blood cell production and reducing transfusions. Sentinel lymph node mapping using SPIO aids in diagnosing lymph node metastases in uterine cancer. Fe supplements may indicate rather than cause endometrial cancer, as tumor tissues show higher Fe levels than healthy tissues, potentially influenced by iron’s role in HIF-1α activation and NF-kB pathway in endometrial cancer cells.	[140,141,142,143,144]

**Table 2:** Affected pathways and mechanisms of action of chosen micro- and macronutrients.

Substance	Mechanisms of Action	Pathways Affected	Impact on Pathway
Magnesium	Enhances oxidative stress response, impacts cell cycle, influences DNA repair, regulates cell division	MAPK signaling pathway, cell cycle, oxidative stress pathways	Enhances cell protection mechanisms and cellular stress response
Sodium Butyrate (NaBu)	Induces ferroptosis, affects gene expression and protein acetylation, reverses EMT	Ferroptosis pathway, cell cycle, gene expression pathways	Activates apoptosis and affects gene expression related to cancer
Arsenic Trioxide (ATO)	Inhibits proliferation, induces apoptosis, reduces telomerase activity	Cell proliferation pathways, apoptosis pathways, anti-estrogenic activity	Inhibits growth and induces cell death in cancer cells
Cadmium (Cd^2+^)	Increases oxidative stress, inhibits DNA repair, suppresses apoptosis, mimics hormones	Oxidative stress pathways, apoptosis pathways	Disrupts cellular protection mechanisms and mimics hormonal effects
Sodium Selenite	Induces oxidative stress, affects antioxidant proteins, leads to apoptosis, increases ROS production, reduces mitochondrial membrane permeability	Oxidative stress pathways, apoptosis pathways	Induces cell death through oxidative stress and mitochondrial damage
Selenium Nanoparticles (SeNPs)	Enhances ROS production, induces apoptosis, affects mitochondrial function, induces cell cycle arrest, DNA fragmentation, enhances targeting of cancer cells	Apoptosis pathway, mitochondrial dysfunction, photothermal therapy	Enhances apoptosis and induces cell death through ROS and PTT
PEGylated Calcium Sulfide Nanoparticles (MCSP)	Induces pyroptosis, activates STING pathway, enhances anti-tumor immune response, inhibits tumor growth	STING signaling pathway, pyroptosis pathway	Activates immune response and enhances tumor targeting
Cobalt (III) Schiff Base Complex (M3)	Blocks DNA synthesis, reduces specific protein levels, activates various pathways; disrupts nuclear division, induces apoptosis	ER stress, apoptosis, cell cycle, DNA damage pathways	Disrupts cell division and activates apoptosis pathways

**Table 3:** Association between trace elements and endometrial cancer.

Trace Element	Result	References
Copper	Cu acts as a cofactor for SOD, protecting against free radicals, but its carcinogenic properties stem from ROS that damage DNA and promote angiogenesis in tumors. Cancer cells exhibit elevated copper levels crucial for angiogenesis. Targeting Cu with specific chelating agents can be an effective anti-cancer approach. Copper-containing IUDs may reduce the risk of endometrial and cervical cancers. Cu also plays a role in cancer treatment, with Cu/TiO_2_ nanoparticles and CTR1 enhancing the efficacy of drugs like cisplatin.	[145,148,151,152,153]
Chromium	Cr release assays, which measure cytotoxicity, were used to evaluate the effectiveness of sacituzumab govitecan (SG), an antibody-drug conjugate targeting Trop-2, in endometrial cancer cell lines, demonstrating significant tumor growth inhibition in poorly differentiated, chemotherapy-resistant xenografts. In USC siRNA targeted to cancer cells could significantly enhance therapeutic efficacy. Research shows that serum Cr levels are higher in patients with uterine fibroids.	[155,157,159]
Selenium	Se enhances immune cell activity, acts as a vital component of antioxidants like GPx, participates in redox signaling and thyroid hormone metabolism. Selenium, delivered through food or supplements, is converted into selenoproteins which protect against oxidative stress and support the immune system. Selenium’s anticancer properties are linked to its role in selenoproteins and its ability to induce oxidative stress in cancer cells, leading to apoptosis. Selenium nanoparticles and compounds like sodium selenite have shown promise in inducing cancer cell death and enhancing the efficacy of existing cancer therapies. Se levels in blood and tissues can serve as markers for cancer prognosis, with lower Se levels associated with higher cancer risk and poor outcomes.	[39,160,161,162,163,164,165,166,167,168,169]
Boron	B plays a potential role in combating endometrial cancer through its impact on the ECC-1 human endometrial adenocarcinoma cell line. A study found that a binuclear boron–fluoride complex significantly inhibited ECC-1 cell growth by damaging DNA and inducing apoptosis. Research in Türkiye indicated that higher dietary B intake correlated with a reduced incidence of cervical cancer. This suggests that boron could interfere with the life cycle of HPV, thereby reducing cancer occurrence.	[171,172]
Manganese	Mn plays a role in endometrial cancer through its involvement in oxidative stress reactions as a cofactor of MnSOD. Studies show higher Mn levels in endometrial cancer tissues, though serum Mn is not a risk factor. MnSOD helps mitigate ROS in mitochondria. Genetic polymorphisms in MnSOD can influence cancer risk, with some genotypes offering protection when antioxidant levels are adequate. Mn compounds are also used in enhancing radiation therapy and immunotherapy, by improving oxygen levels and stimulating immune responses.	[174,175,176,177,178,179]
Cobalt	Cobalt-60 is used in high-dose rate brachytherapy for treating cervical cancer. It has shown effectiveness comparable to iridium-192 and is cost-effective due to its long half-life. Cobalt ferrite nanoparticles exhibit potential in cancer treatment by altering cellular metabolism and reducing tumor growth-related metabolites in HeLa cells. Inhalation of Co in an industrial setting can lead to health risks.. Elevated cobalt levels are observed in individuals with uterine fibroids, which may act as reservoirs for the metal, influencing systemic exposure and potentially impacting fibroid growth through interactions with estrogen receptors.	[140,182,184,185]
Iodine	Iodine’s impact on endometrial cancer is uncertain. Earlier hypotheses suggested geographic cancer rate variations might inversely relate to I intake, but recent data from sub-Saharan Africa challenge this. Low I intake theoretically increases gonadotrophin stimulation, potentially leading to a hyperestrogenic state with elevated estrone and estradiol levels.	[1,186,187]
Fluoride	F impacts oxidative stress pathways, promoting DNA damage and potentially carcinogenesis. F alters antioxidant enzymes like SOD and catalase, affects cell signaling related to proliferation and apoptosis, and influences biomarkers of oxidative stress. Research highlights fluoride’s potential in treating endometrial cancer through novel compounds like [L(BF_2_)_2_], which induce oxidative stress, DNA damage, and apoptosis in cancer cells.	[171,188,189]
Zinc and copper	Zn and Cu are essential for reproductive health as antioxidants. Zn regulates detoxification through Nrf2 and boosts antioxidant defenses with Cu/Zn-SOD. Cu aids enzymes like SOD3. Low levels of Zn and Cu in serum are linked to higher risk of endometrial cancer, suggesting their role in cancer development and prognosis.	[14,123,199,200,201]
Arsenic	Arsenic trioxide (ATO) shows promise in treating endometrial cancer by inhibiting cell proliferation, reducing telomerase activity, and inducing apoptosis in cancer cells. It also exhibits anti-estrogenic effects through the MAPK signaling pathway. Studies suggest ATO’s potential in treating platinum-resistant recurrent endometrial cancer, expanding its role beyond leukemia to solid tumors.	[202,203,204,205]
Cadmium	Cd exposure is linked to an increased risk of endometrial cancer. Studies show a positive correlation between Cd exposure and the onset of endometrial cancer, with elevated Cd levels associated with higher risks. Cd induces oxidative stress, inhibits DNA repair mechanisms, and suppresses apoptosis through various pathways. It may also act as a metallohormone, mimicking estrogen and potentially promoting the development of endometrial cancer. Dietary intake of Cd above certain levels has been associated with decreased overall survival in endometrial cancer patients.	

## Data Availability

No new data were created or analyzed in this study. Data sharing is not applicable to this article.

## Abbreviations

BMI	body mass index
HPV	human papillomavirus
LNs	lymph nodes
MnSOD	manganese superoxide dismutase
OS	overall survival
PMB	postmenopausal bleeding
ROS	reactive oxygen species
SLN	sentinel lymph node
TF	tissue factor
SPIO	superparamagnetic iron oxide
SOD	superoxide dismutase
